# Dynamical machine learning volumetric reconstruction of objects’ interiors from limited angular views

**DOI:** 10.1038/s41377-021-00512-x

**Published:** 2021-04-07

**Authors:** Iksung Kang, Alexandre Goy, George Barbastathis

**Affiliations:** 1 Department of Electrical Engineering and Computer Science, Massachusetts Institute of Technology, 77 Massachusetts Ave, Cambridge, MA USA; 2 Department of Mechanical Engineering, Massachusetts Institute of Technology, Cambridge, MA 02139 USA; 3 Singapore-MIT Alliance for Research and Technology (SMART) Centre, 1 Create Way, Singapore, 117543 Singapore; 4 Present Address: Omnisens SA, Morges, 1110 Switzerland

**Keywords:** Imaging and sensing, Applied optics

## Abstract

Limited-angle tomography of an interior volume is a challenging, highly ill-posed problem with practical implications in medical and biological imaging, manufacturing, automation, and environmental and food security. Regularizing priors are necessary to reduce artifacts by improving the condition of such problems. Recently, it was shown that one effective way to learn the priors for strongly scattering yet highly structured 3D objects, e.g. layered and Manhattan, is by a static neural network [Goy et al. *Proc. Natl. Acad. Sci*. 116, 19848–19856 (2019)]. Here, we present a radically different approach where the collection of raw images from multiple angles is viewed analogously to a dynamical system driven by the object-dependent forward scattering operator. The sequence index in the angle of illumination plays the role of discrete time in the dynamical system analogy. Thus, the imaging problem turns into a problem of nonlinear system identification, which also suggests dynamical learning as a better fit to regularize the reconstructions. We devised a Recurrent Neural Network (RNN) architecture with a novel Separable-Convolution Gated Recurrent Unit (SC-GRU) as the fundamental building block. Through a comprehensive comparison of several quantitative metrics, we show that the dynamic method is suitable for a generic interior-volumetric reconstruction under a limited-angle scheme. We show that this approach accurately reconstructs volume interiors under two conditions: weak scattering, when the Radon transform approximation is applicable and the forward operator well defined; and strong scattering, which is nonlinear with respect to the 3D refractive index distribution and includes uncertainty in the forward operator.

## Introduction

Optical tomography reconstructs the three-dimensional (3D) internal refractive index profile by illuminating the sample at several angles and processing the respective raw intensity images. The reconstruction scheme depends on the scattering model that is appropriate for a given situation. If the rays through the sample can be well approximated as straight lines, then the accumulation of absorption and phase delay along the rays is an adequate forward model, i.e. the projection or Radon transform approximation applies. This is often the case with hard x-rays through most materials including biological tissue; for that reason, Radon transform inversion has been widely studied^1–10^. The problem becomes even more acute when the range of accessible angles around the object is restricted, a situation that we refer to as “limited-angle tomography,” due to the missing cone problem^11–13^.

The next level of complexity arises when diffraction and multiple scattering must be taken into account in the forward model; then, the Born or Rytov expansions and the Lippmann-Schwinger integral equation^14–18^ are more appropriate. These follow from the scalar Helmholtz equation using different forms of expansion for the scattered field^19^. In all these approaches, weak scattering is obtained from the first order in the series expansion. Holographic approaches to volumetric reconstruction generally rely on this first expansion term^20–31^. Often, solving the Lippmann-Schwinger equation is the most robust approach to account for multiple scattering, but even then, the solution is iterative and requires excessive amount of computation especially for complex 3D geometries. The inversion of these forward models to obtain the refractive index in 3D is referred to as inverse scattering, also a well-studied topic^32–39^.

An alternative to the integral methods is the Beam Propagation Method (BPM), which sections the sample along the propagation distance z into slices, each slice scattering according to the thin transparency model, and propagates the field from one slice to the next through the object^40^. Despite some compromise in accuracy, BPM offers comparatively light load of computation and has been used as forward model for 3D reconstructions^18^. The analogy of the BPM computational structure with a neural network was exploited, in conjunction with gradient descent optimization, to obtain the 3D refractive index as the “weights” of the analogous neural network in the learning tomography approach^41–43^. BPM has also been used with more traditional sparsity-based inverse methods^33,44^. Later, a machine learning approach with a Convolutional Neural Network (CNN) replacing the iterative gradient descent algorithm exhibited even better robustness to strong scattering for layered objects, which match well with the BPM assumptions^45^. Despite great progress reported by these prior works, the problem of reconstruction through multiple scattering remains difficult due to the extreme ill-posedness and uncertainty in the forward operator; residual distortion and artifacts are not uncommon in experimental reconstructions.

Inverse scattering, as inverse problems in general, may be approached in a number of different ways to regularize the ill-posedness and thus provide some immunity to noise^46,47^. Recently, thanks to a ground-breaking observation from 2010 that sparsity can be learnt by a deep neural network^48^, the idea of using machine learning to approximate solutions to inverse problems also caught on ref. ^49^. In the context of tomography, in particular, deep neural networks have been used to invert the Radon transform^50^ and recursive Born model^32^, and were also the basis of some of the papers we cited earlier on holographic 3D reconstruction^28–30^, learning tomography^41–43^, and multi-layered strongly scattering objects^45^. In prior work on tomography using machine learning, generally, the intensity projections are all fed simultaneously as inputs to a computational architecture that includes a neural network, and the output is the 3D reconstruction of the refractive index. The role of the neural network is to learn (1) the priors that apply to the particular class of objects being considered and (2) the relationship of these priors to the forward operator (Born, BPM, etc.) so as to produce a reasonable estimate of the inverse.

Here we propose a rather distinct approach to exploit machine learning for a generic 3D refractive index reconstruction independent of the type of scattering. Our motivation is that, as the angle of illumination is changed, the light goes through *the same scattering volume*, but the scattering events, weak or strong, follow a different sequence. At the same time, the raw image obtained from a new angle of illumination adds information to the tomographic problem, but that information is constrained by (i.e. is not orthogonal to) the previously obtained patterns. We interpret this as similar to a dynamical system, where the output is constrained by the history of earlier inputs as time evolves and new inputs arrive. (The convolution integral is the simplest and best-known expression of this relationship between the output of a system and the history of the system’s input.) An alternative interpretation is as dynamic programming^51^, where the system at each step reacts so as to incrementally improve an optimality criterion—in our case, the reconstruction error metric.

**Fig. 1:**
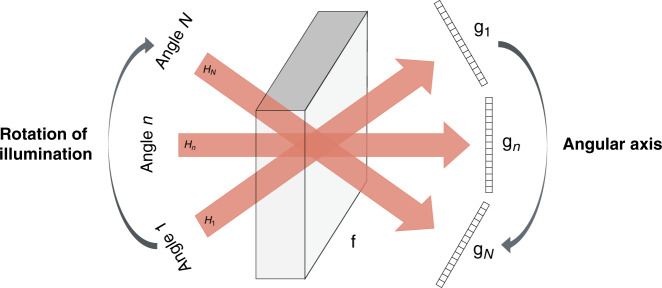
Definition of the angular axis according to illumination angles. Each angle of illumination, here labeled as angular axis, corresponds to a time step in an analogous temporal axis

The analogy between tomography and a dynamical system suggests the RNN architecture as a strong candidate to process raw images in sequence, as they are obtained one after the other; and process them recurrently so that each raw image from a new angle improves over the reconstructions obtained from the previous angles. Thus, we treat multiple raw images under different illumination angles as a temporal sequence, as shown in Fig. 1. The angle index replaces what is a dynamical system would have been the time *t*. This idea is intuitively appealing; it also leads to considerable improvement in the reconstructions, removing certain artifacts that were visible in the strong scattering case of ref. ^45^.

The dynamic reconstruction methodology applies, for example, too weak scattering where the raw images are the sinograms; and too strong scattering, where the raw images are better interpreted as intensity diffraction patterns. The purpose of the learning scheme is to augment this relationship with regularization priors applicable to a certain class of objects of interest.

The way we propose to use RNNs in this problem is quite distinct from the recurrent architecture proposed first in ref. ^48^ and subsequently implemented, replacing the recurrence by a cascade of distinct neural networks, in refs. ^50,52,53^, among others. In these prior works, the input to the recurrence can be thought of as clamped to the raw measurement, as in the proximal gradient^54^ and related methods; whereas, in our case, the input to the recurrence is itself dynamic, with the raw images from different angles forming the input sequence. Moreover, by utilizing a modified gated recurrent unit (more on this below) rather than a standard neural network, we do not need to break the recurrence up into a cascade.

Typical applications of RNNs^55,56^ are in temporal sequence learning and identification. In imaging and computer vision, RNN is applied in 2D and 3D: video frame prediction^57–60^, depth map prediction^61^, shape inpainting^62^; and stereo reconstruction^63,64^ or segmentation^65,66^ from multi-view images, respectively. Stereo, in particular, bears certain similarities to our tomographic problem here, as sequential multiple views can be treated as a temporal sequence. To establish the surface shape, the RNNs in these prior works learn to enforce consistency in the raw 2D images from each view and resolve the redundancy between adjacent views in recursive fashion through the time sequence (i.e. the sequence of view angles). Non-RNN learning approaches have also been used in stereo, e.g. Gaussian mixture models^67^. In computed tomography, in particular, an alternate dynamical neural network of the Hopfield type has been used successfully^68^.

In this work, we replaced the standard Long Short-Term Memory (LSTM)^56^ implementation of RNNs with a modified version of the newer Gated Recurrent Unit (GRU)^69^. The GRU has the advantage of fewer parameters but generalizes comparably with the LSTM. Our GRU employs a separable-convolution scheme to explicitly account for the asymmetry between the lateral and axial axes of propagation. We also utilize an angular attention mechanism whose purpose is to learn how to reward specific angles in proportion to their contribution to reconstruction quality^70^. We found that for the strongly anisotropic samples or scanning schemes the angular attention mechanism is effective.

The results of our simulation and experimental study on a generic interior volumetric reconstruction are in Results. We first show numerically that the dynamical machine learning approach is suitable to tomographic reconstruction under more restrictive and commonly used Radon transform assumption, i.e. weak scattering. Then, we demonstrate the applicability of the dynamical approach to strong scattering tomography. We show significant improvement over static neural network-based reconstructions of the same experimental data under the strong scattering assumption. The improvement is shown both visually and in terms of several quantitative metrics. Results from an ablation study indicate the relative significance of the new components we introduced to the quality of the reconstructions.

## Results

Our first investigation of the recurrent reconstruction scheme is for weak scattering, i.e. when the Radon transform approximation applies and with a limited range of available angles. For simulation, each sample consists of random number, between 1 and 5 with equal probability, of ellipsoids at random locations with arbitrarily chosen sizes, amplitudes, and angles, thus spatially isotropic in average. Rotation is applied along the *x*-axis, from −10° to +10° with 1° increment, thus 21 projections per sample under a parallel-beam geometry. The Filtered Backprojection (FBP) algorithm^3^ is used to generate crude estimates from the projections. *n*th FBP Approximant (*n* = 1,2,…,21) is the reconstruction by the FBP algorithm using *n* projections of *n* angles starting from −10°.

**Fig. 2:**
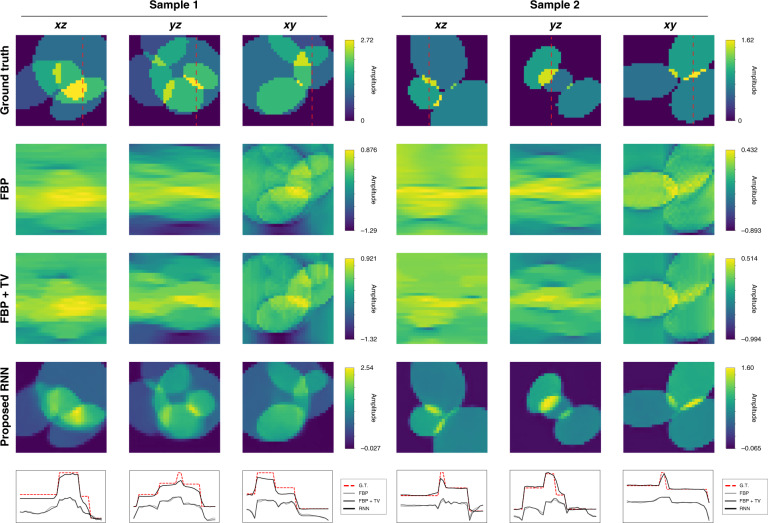
Dynamic approach for projection tomography (i.e. subject to the Radon transform approximation) using simulated data. Each simulated sample contains randomly generated ellipsoids. Shown are *xz*, *yz*, and *xy* cross-sections of ground truth and reconstructions by Filtered Backprojection (FBP), TV-regularized Filtered Backprojection (FBP + TV), and the proposed RNN. Red dashed lines show where the 1D cross-section profiles are drawn

**Fig. 3:**
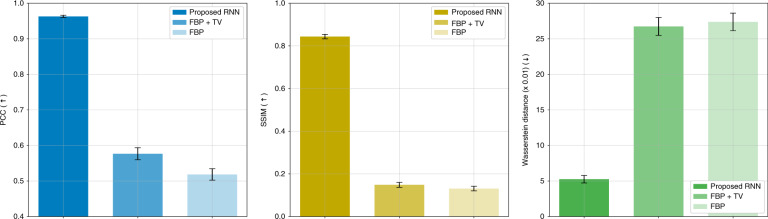
Test performance under the weak scattering condition. The figure quantitatively compares the recurrent scheme with FBP + TV, and FBP. The graphs show the means and 95% confidence intervals

The reconstructions by the RNN are compared in Fig. 2 with FBP and Total Variation (TV)-regularized reconstructions using TwIST^71^ for qualitative and visual comparison. Here a TV-regularization parameter is set to be 0.01, and the algorithm is run up to 200 iterations until its objective function saturates. Figure 3 shows the quantitative comparison on test performance using three different quantitative metrics, where FBP and FBP + TV yielded much lower values than the recurrent scheme.

**Fig. 4:**
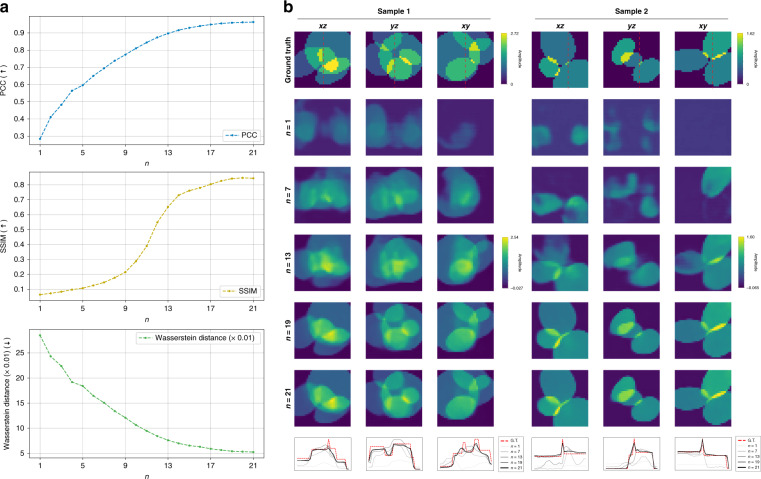
Progression of 3D reconstruction performance under the weak scattering condition. **a** Three quantitative metrics are used to quantify test performance of the trained recurrent neural network under the weak scattering assumption using simulated data, i.e. Pearson Correlation Coefficient (PCC), Structural Similarity Index Metric (SSIM), and Wasserstein distance. **b** Progression of 3D reconstruction performance as the FBP Approximants $n = 1,\, \ldots ,\,N( = 21)$ are projected to the recurrent scheme. Movies as Supplementary movies 1 and 2 show the progression of the reconstructions of Samples 1 and 2, respectively. Red dashed lines show where the 1D cross-section profiles are drawn

Figure 4 shows the evolution of test reconstructions as new projections or FBP Approximants are presented to the dynamical scheme. When the recurrence starts with *n* = 1, the volumetric reconstruction is quite poor; as more projections are included, the reconstruction improves as expected. It is also interesting to see that not all the angles are needed to achieve reasonable quality of reconstructions as the graphs and reconstructions in Fig. 4a, b saturate around *n* = 19.

Details of the recurrent architecture for the weak scattering assumption are presented in Fig. 16b. To quantify the relative contributions to reconstruction quality of each element in the architecture, the elements one by one are ablated (-) or substituted ($\rightleftarrows$) with alternatives. This ablation study is performed on a strategy to giving weights on hidden features, separable convolution, and ReLU (rectified linear unit) activation function^72^ inside the recurrence cell. Specifically, in the study, (1) an angular attention mechanism is ablated, or only last hidden feature ***h***_*n*_ is taken into account before the decoder instead of the angular attention mechanism; (2) a separable convolution scheme is ablated, thus the standard 3D convolution; and (3) ReLU activation function is ablated and then substituted with the tanh activation function, which is more usual^69^. The ablated architectures are trained under the same training scheme (for more details, see Training the recurrent neural network in Materials and methods) and tested with the same simulated data.

**Fig. 5:**
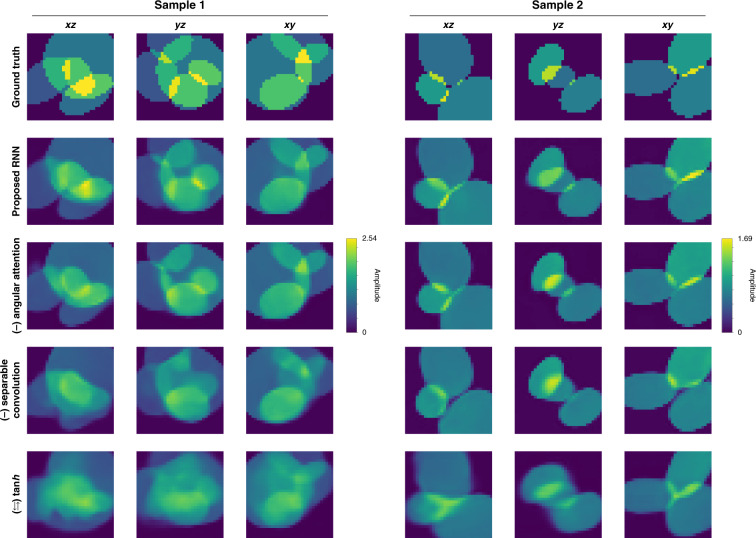
Ablation study on the recurrent architecture under the weak scattering condition. Qualitative representations are shown when some elements of the recurrent architecture in Fig. 16b are ablated, and the rows are ordered by increasing severity of the ablation effects according to Fig. 6

**Fig. 6:**
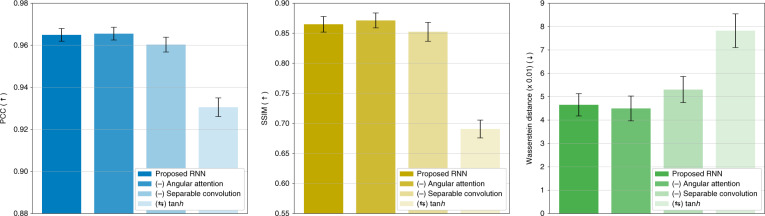
Quantitative analysis on test performance in the ablation study under the weak scattering condition. The graphs show the means and 95% confidence intervals

Visually, in Fig. 5, test performance largely degrades as the ablation happens on the ReLU activation function and separable convolution, which is also found quantitatively in Fig. 6. Therefore, for the Radon case, we find that (1) ReLU activation function is highly desirable instead of the native tanh function; (2) the separable convolution is helpful when designing a recurrent unit and encoder/decoder for tomographic reconstructions under the weak scattering assumption. However, for the ablation of the angular attention mechanism, it makes no large difference from the performance of the proposed model, which is because the objects used for training and testing are isotropic in average and do not show any preferential direction.

Here, we perform an additional study on the role of the angular attention mechanism by granting a preferential direction or spatial anisotropy to the objects of interest. In this study, the mean major axis of the objects is assumed to be parallel to the *z-*axis, i.e. the objects are elongated along the axis. Instead of the previously examined symmetric angular scanning range (−10°, +10°), we now consider the asymmetric case (−15°, +5°).

**Fig. 7:**
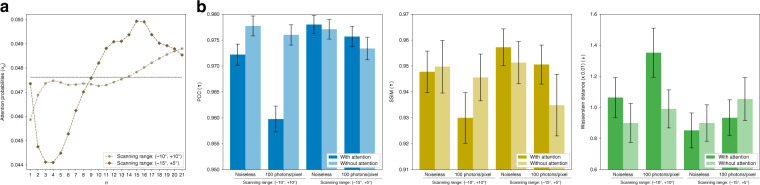
Attention probabilities and angular attention mechanism for different ranges of scanning. **a** Attention probabilities according to different ranges of scanning. Here, for the scanning range of (−10°, +10°), *n* = 1 and 21 correspond to −10° and +10°, respectively; and for the scanning range of (−15°, +5°), they correspond to −15° and +5°, respectively. **b** Test performance for our examined scanning ranges with and without the angular attention mechanism. The graphs show the means and 95% confidence intervals

In Fig. 7a, the angular attention with the asymmetry now gives attention on *n* differently, i.e. its peak translated to the left by 5. It is because the projections from higher angles may contain more useful information on the objects due to the directional preference of the objects, thus the distribution of the attention probabilities is now attracted to the lower indices. In Fig. 7b, we quantitatively show that the angular attention improves performance in the case of the asymmetric scanning of objects with directionality regardless of the noise present in projections.

**Fig. 8:**
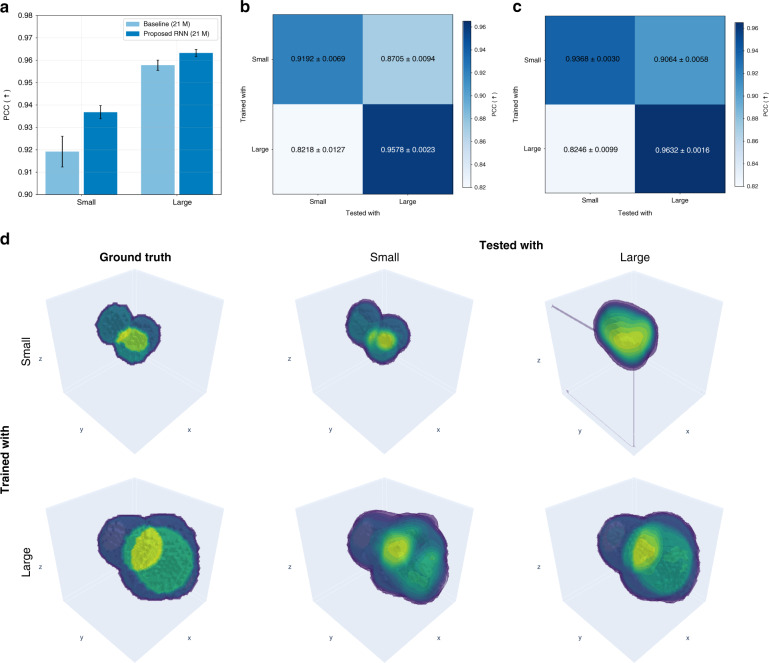
Sample feature size and testing performance. **a** Quantitative comparison of testing performance in terms of feature size, and cross-domain generalization result of (**b**) the baseline model (21 M) and (**c**) the proposed RNN (21 M). **d** Qualitative comparison of a volumetric reconstruction for different cases of training and testing conditions in terms of feature size (see Table S1 in Supplementary Information (SI) Section S1 for more details)

**Fig. 9:**
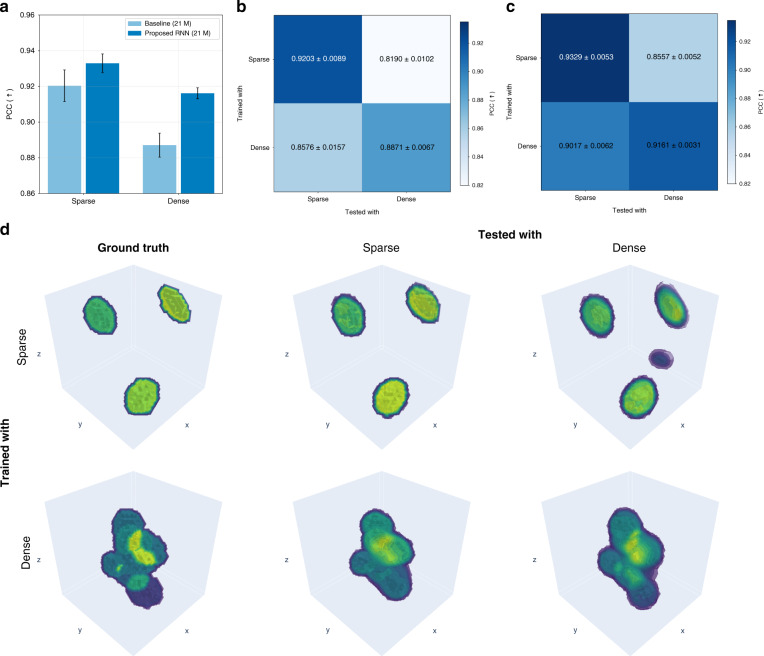
Sample sparsity and testing performance. **a** Quantitative comparison of testing performance in terms of sparsity, and cross-domain generalization result of (**b**) the baseline model (21 M) and (**c**) the proposed RNN (21 M). **d** Qualitative comparison of a volumetric reconstruction for different cases of training and testing conditions in terms of sparsity. For more details, see Table S1 in SI Section S1

Figures 8 and 9 characterize our proposed method in terms of feature size and feature sparsity, as well as cross-domain generalization, compared to the baseline model (see Training the recurrent neural network in “Materials and methods” for details). Networks trained with examples with small and dense features tend to generalize better and with less artifacts than large and sparse features, in agreement with ref. ^73^. Lastly, and not surprisingly, overall reconstruction quality is better when feature size is large and features are sparse.

**Fig. 10:**
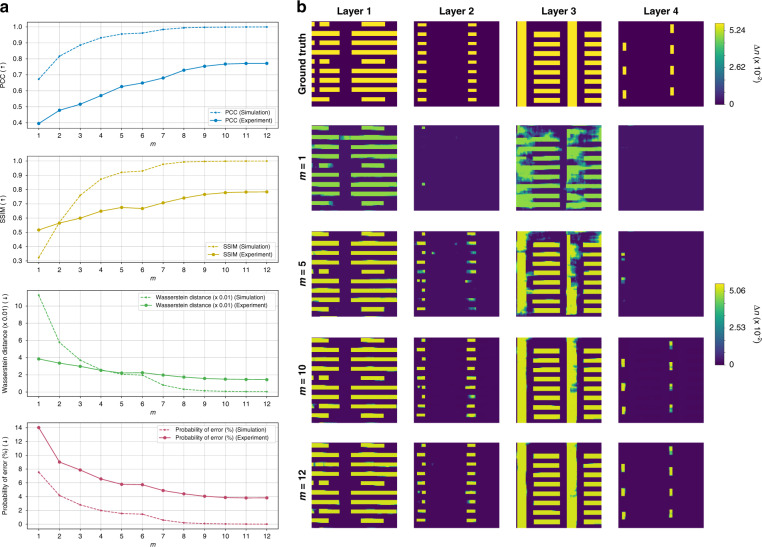
Progress of 3D reconstruction performance as diffraction patterns from different angles are presented to the recurrent scheme. Here, DWMA Approximants ${\tilde{\boldsymbol f}}_m^{[1]}\left( {m = 1,\,2,\, \ldots ,\,12} \right)$ are presented to the RNN sequentially. **a** Four quantitative metrics (PCC, SSIM, Wasserstein distance, and Probability of Error; PE) quantitatively show the progress using both simulated and the experimental data. The graphs show the mean of the metrics of Layers 1–4. **b** Qualitative comparison using experimental data with different *m* = 1, 5, 10, and 12

Next, we investigate the case when the Radon transform is not applicable, i.e. tomography under strong scattering conditions and under a similarly limited-angle scheme. The RNN is first trained with the single-pass, gradient descent-based Approximants Eq. (4) of simulated diffraction patterns (see Training and testing procedures in Materials and methods), and then tested with the simulated ones and additionally with the TV-based Approximants Eq. (5) of experimentally obtained diffraction patterns. TV regularization is only applied to the experimental patterns. To reconcile any experimental artifacts, there is an additional step of Dynamic Weighted Moving Average (DWMA) on the Approximants ${\boldsymbol{f}}_n^{[1]}(n = 1,\,2,\, \ldots ,\,42)$, hence DWMA Approximants ${\tilde{\boldsymbol f}}_m^{[1]}(m = 1,\,2,\, \ldots ,\,12)$. See “Materials and methods” for more details in the DWMA process. The evolution of the RNN output as more DWMA Approximants are presented is shown in Fig. 10 and shows a similar improvement with recurrence *m* as in the Radon case of Fig. 4. Also, like the Radon case, it is interesting to see that not all the Approximants are needed to acquire reasonable quality of reconstructions: the graphs in Fig. 10a saturate around *m* = 10 and the visual quality of the reconstructions at *m* = 10–12 in Fig. 10b does not largely differ.

**Fig. 11:**
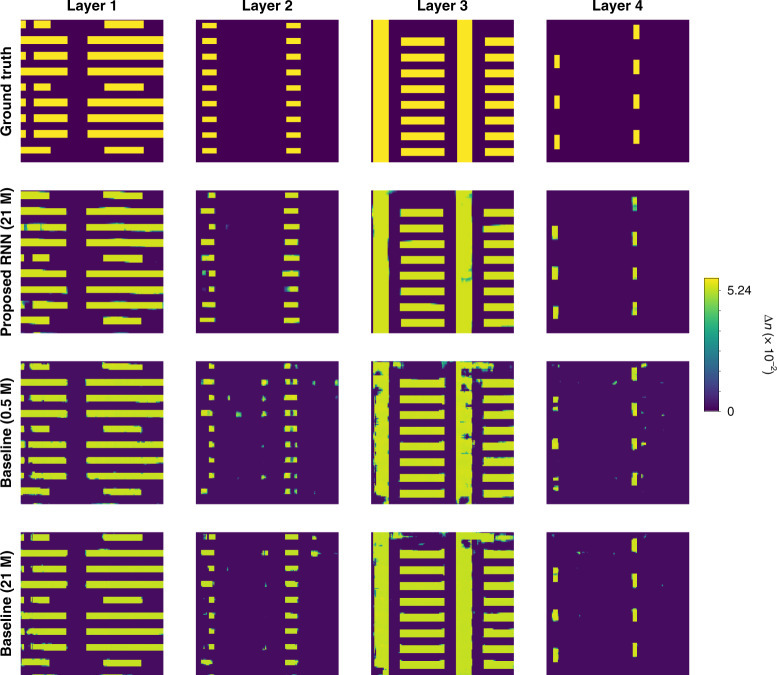
Qualitative comparison on test performance between the baseline and proposed architectures using experimental data. The baseline architectures are 3D-DenseNet CNN architectures with 0.5 M and 21 M parameters. Our proposed architecture is a recurrent neural network with elements (for more details, see Computational architecture in Materials and methods)

**Fig. 12:**
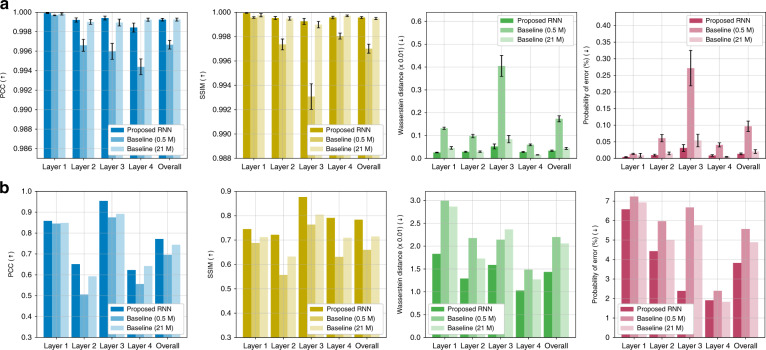
Test performance on simulated and experimental data under the strong scattering condition. For the quantitative comparison, four different metrics are used, i.e. PCC, SSIM, Wasserstein distance, and PE on (**a**) simulated and (**b**) experimental data. Graphs in (**a**) show the means and 95% confidence intervals. Raw data of Fig. 12b can be found in Table S2 in SI Section S2

For comparison, the 3D-DenseNet architecture with skip connections in ref. ^45^ and its modified version with more parameters to match with that of our RNN are set as baseline models (see Training the recurrent neural network in Materials and methods for details). Our RNN has approximately 21 M parameters, and visual comparisons with the baseline 3D-DenseNets with 0.5 M and 21 M parameters are shown in Fig. 11. The RNN results show substantial visual improvement, with fewer artifacts and distortions compared to the static approaches of ref. ^45^, even when the number of parameters in the latter matches ours. PCC, SSIM, Wasserstein distance, and PE are used to quantify test performance using simulated and experimental data in Fig. 12.

We also conducted an ablation study of the learning architecture of Fig. 16d. Similar to the Radon case, each component in the architecture was ablated or substituted with its alternative, one at a time: (1) ReLU was ablated and then substituted with the native tanh activation function, (2) the separable convolution was ablated, thus the standard 3D convolution, and (3) the angular attention mechanism was ablated, or only the last hidden feature was given attention. The ablated architectures are also trained under the same training scheme (see Training the recurrent neural network in “Materials and methods” for more details) and tested with both the simulated Eq. (4) and experimental Approximants Eq. (5).

**Fig. 13:**
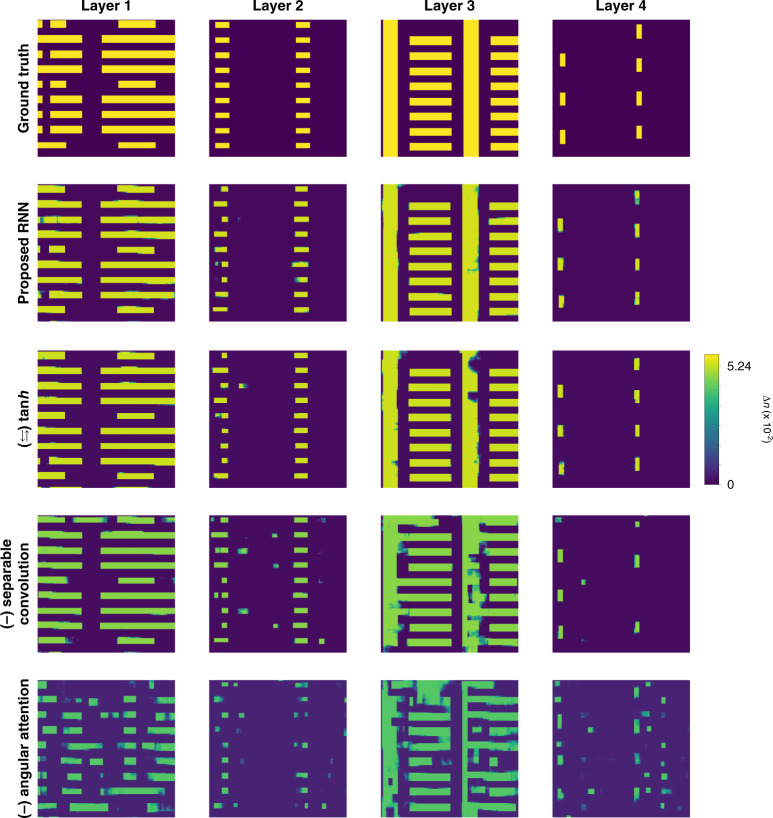
Visual quality assessment from the ablation study on elements (see “Computational architecture in Materials and methods” for details) using experimental data. Rows 3–5 show reconstructions based on experimental data for each layer upon the ablation and substitution of ReLU activation in Eq. (10) with the more common tanh activation function instead (row 3); ablating the separable convolution scheme, thus the standard 3D convolution (row 4); ablating the angular attention mechanism and putting attention to only last hidden feature (row 5). The rows are ordered by increasing severity of the ablation effect according to Fig. 14b

**Fig. 14:**
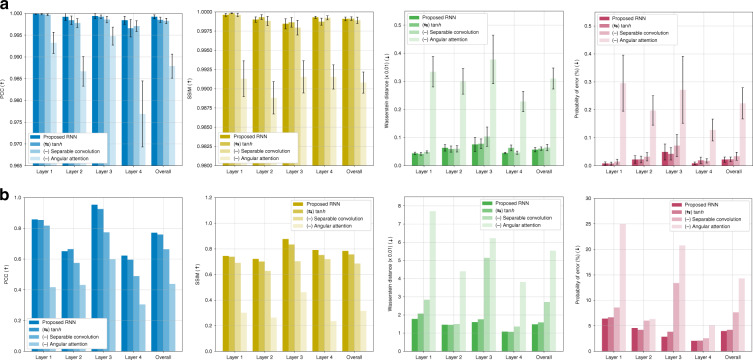
Ablation study on the recurrent architecture under the strong scattering condition. Quantitative assessment from the ablation study using four different metrics on (**a**) simulated and (**b**) experimental data. Graphs in (**a**) show the means and 95% confidence intervals. Raw data of Fig. 14b can be found in Table S3 in SI Section S2

Visually in Fig. 13, unlike the Radon case, paying attention only to the last hidden feature affects and degrades the testing performance worst. Also, it is important to note that the ablation of the separable convolution scheme brings degradation in test performance according to Fig. 13. The decrease in test performance by the substitution of ReLU with the more common tanh is comparatively marginal. These findings are supported quantitatively as well in Fig. 14.

Thus, under the strong scattering condition, we find that (1) hidden features from all angular steps need to be taken into consideration with the angular attention mechanism for reconstructions to get a better test performance although the last hidden feature is assumed to be informed of the history of the previous angular steps; (2) replacing the standard 3D convolution with the separable convolution helps when designing a recurrent unit and a convolutional encoder/decoder for tomographic reconstructions; and (3) the substitution of tanh with ReLU is still useful but may be application dependent.

## Discussion

We have proposed a new recurrent neural network scheme for a generic interior-volumetric reconstruction by processing raw inputs from different angles of illumination dynamically, i.e. as a sequence, with each new angle improving the 3D reconstruction. We found this approach to work well under two types of scattering assumptions: weak (Radon transform) and strong. In the second case, in particular, we observed significant qualitative and quantitative improvement over the static machine learning scheme of ref. ^45^, where the raw inputs from all angles are processed at once by a neural network.

Through the ablation studies, we found that sandwiching the recurrent structure with some key elements between a convolutional encoder/decoder helps improve the reconstructions. We found that the angular attention mechanism takes an important role especially when the objects of interest are spatially anisotropic and performs better than placing all the attention on only the last hidden feature. Even though the last hidden feature is a nonlinear multivariate function of all the previous hidden features, as it has a propensity to reward the latter representations but the former ones^74^, the last hidden feature may not sufficiently represent all angular views. Hence, the angular attention mechanism adaptively merges information from all angles. This is particularly important for our strong scattering case as each DWMA Approximant involves a diffraction pattern of a certain illumination angle; whereas an FBP Approximant under the weak scattering case is computed from several projections in a cumulative fashion.

In addition, interestingly, the relative contributions of other elements, e.g. the separable convolution scheme and ReLU activation, differ in weak and strong scattering assumptions. The substitution of the ReLU with a more common tanh activation brings forth more severe degradation of performance under the weak scattering assumption. Thus, we suggested different guidelines for each scattering assumption.

Lastly, alternative implementations of the RNN could be considered. Examples are LSTMs, Reservoir Computing^75–77^, separable convolution or DenseNet variants for the encoder/decoder and dynamical units. We leave these investigations to future work.

## Materials and methods

### Experiment

**Fig. 15:**
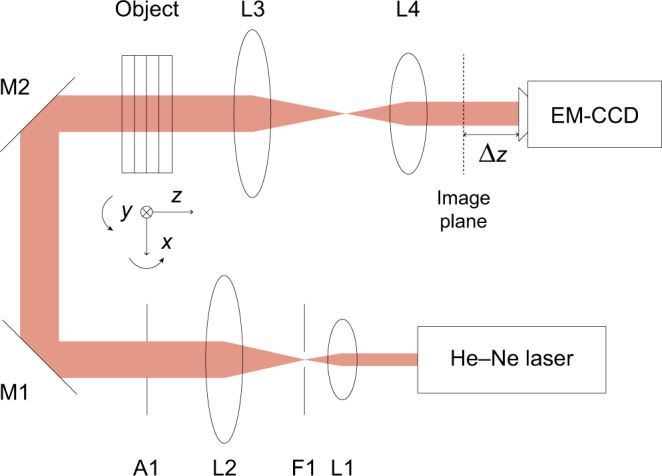
Optical apparatus used for experimental data acquisition^45^. L1-4: lenses, F1: pinhole, A1: aperture, EM-CCD: Electron-Multiplying Charge Coupled Device. $f_{L_3}:f_{L_4} = 2:1$. The object is along both *x* and *y* axes. The defocus distance between the conjugate plane to the exit object surface and the EM-CCD is Δ*z* = 58.2 mm

For the experimental study under the strong scattering assumption, the experimental data are the same as in ref. ^45^, whose experimental apparatus is summarized in Fig. 15. We repeat the description here for the readers’ convenience. The He-Ne laser (Thorlabs HNL210L, power: 20 mW, *λ* = 632.8 nm) illuminated the sample after spatial filtering and beam expansion. The illumination beam was then de-magnified by the telescope ($f_{L_3}:f_{L_4} = 2:1$), and the EM-CCD (Rolera EM-C2, pixel pitch: 8 *μ*m, acquisition window dimension: $1002 \times 1004$) captured the experimental intensity diffraction patterns. The integration time for each frame was 2 ms, and the EM gain was set to × 1. The optical power of the laser was strong enough for the captured intensities to be comfortably outside the shot-noise limited regime.

Each layer of the sample was made of fused silica slabs (*n* = 1.457 at 632.8 nm and at 20 °C). Slab thickness was 0.5 mm, and patterns were carefully etched to the depth of 575 ± 5 nm on the top surface of each of the four slabs. To reduce the difference between refractive indices, gaps between adjacent layers were filled with oil (*n* = 1.4005 ± 0.0002 at 632.8 nm and at 20 °C), yielding binary-phase depth of −0.323 ± 0.006 rad. The diffraction patterns used for training were prepared with simulation precisely matched to the apparatus of Fig. 15. For testing, we used a set of diffraction patterns that was acquired both through simulation (see Approximant computations in Materials and methods for details) and experiment.

For the strong scattering case, objects used for both simulation and experiment are dense-layered, transparent, i.e. of negligible amplitude modulation, and of binary refractive index. They were drawn from a database of IC layout segments^45^. The feature depth of 575 ± 5 nm and refractive index contrast 0.0565 ± 0.0002 at 632.8 nm and at 20 °C were such that weak scattering assumptions are invalid and strong scattering has to be necessarily taken into account. The Fresnel number ranged from 0.7 to 5.5 for the given defocus amount Δ*z* = 58.2 mm for the range of object feature sizes.

To implement the raw image acquisition scheme, the sample was rotated from −10° to +10° with a 1° increment along both the *x* and *y* axes, while the illumination beam and detector remained still. This resulted in *N* = 42 angles and intensity diffraction patterns in total. Note that ref. ^45^ only utilized 22 patterns out of with a 2-degree increment along both *x* and *y* axes. The comparisons we show later are still fair because we retrained all the algorithms of ref. ^45^ for the 42 angles and 1° increment.

### Computational architecture

**Fig. 16:**
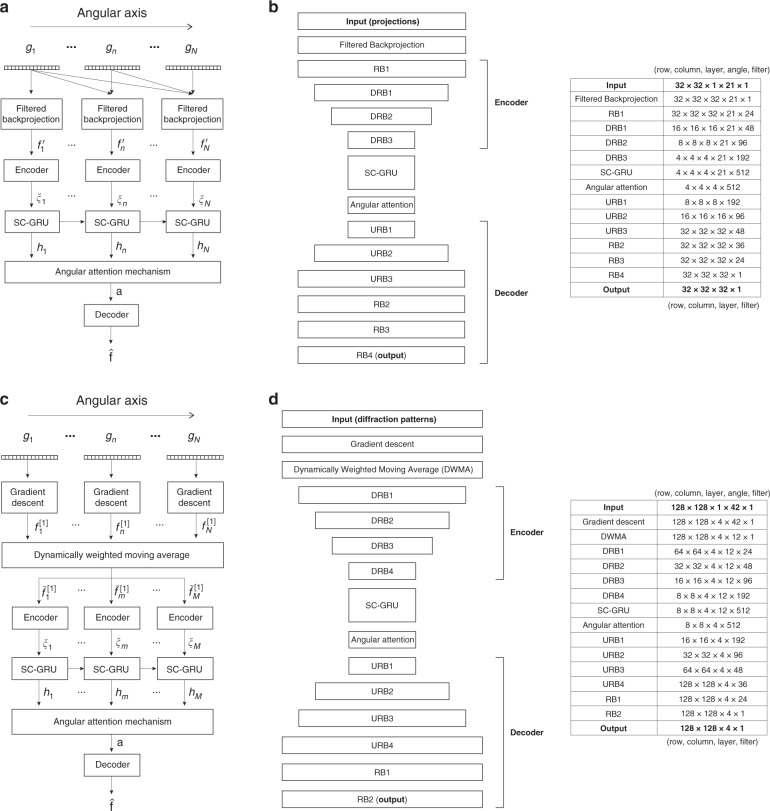
Details on implementing the dynamical scheme. Overall network architecture and tensorial dimensions of each layer for (**a**–**b**) weak scattering and (**c**–**d**) strong scattering cases. (**a**) and (**c**) show unrolled versions of the architectures in (b) and (d), respectively. (a-b) Weak scattering case: at *n*th step, *n* Radon projections ${\boldsymbol{g}}_1,\, \ldots ,\,{\boldsymbol{g}}_n$ create an Approximant ${\boldsymbol{f}}_n^\prime$ by a FBP operation, and a sequence of FBP Approximants ${\boldsymbol{f}}_n^\prime ,\,n = 1, \ldots ,\,N\left( { = 21} \right),$ is followed by an encoder and a recurrent unit. There is an angular attention block before a decoder for the 3D reconstruction ${\hat{\boldsymbol f}}$, (**c**-**d**) Strong-scattering case: the raw intensity diffraction pattern ${\boldsymbol{g}}_n,\,n = 1,\, \ldots ,\,N\,( = 42),$ of the *n*th angular sequence step is followed by gradient descent and the Dynamically Weighted Moving Average (DWMA) operations to construct another Approximant sequence ${\tilde{\boldsymbol f}}_m^{\left[ 1 \right]},\,m = 1, \ldots ,\,M\,( = 12)$ from original Approximants ${\boldsymbol{f}}_n^{\left[ 1 \right]}$. TV regularization is applied to the gradient descent only for experimental diffraction patterns. The DWMA Approximants ${\tilde{\boldsymbol f}}_m^{\left[ 1 \right]}$ are encoded to ***ξ***_m_ and fed to the recurrent dynamical operation whose output sequence ${\boldsymbol{h}}_m,\,m = 1,\, \ldots ,\,12$, and the angular attention mechanism them into a single representation *a*, which is finally decoded to produce the 3D reconstruction ${\hat{\boldsymbol f}}$. For both cases, training adapts the weights of the learned operators in this architecture to minimize the training loss function ${\cal{E}}\left( {{\boldsymbol{f}},{\hat{\boldsymbol f}}} \right)$ between ${\hat{\boldsymbol f}}$ and the ground truth object ***f***

Figure 16 shows the proposed RNN architectures for both scattering assumptions in detail. Details of the forward model and Approximant (pre-processing) algorithm, the separable-convolution GRU, convolutional encoder and decoder, and the angular attention mechanism are described in Materials and methods. The total number of parameters in both computational architectures is ∼ 21 M (more on this topic in Training the recurrent neural network in Materials and methods.).

### Approximant computations

Under the weak scattering condition, amplitude phantoms with the random number between 1 and 5 of ellipsoids with arbitrarily chosen dimensions and amplitude values at random locations are illuminated within a limited-angle range along one axis, thus spatially isotropic in average. The angle is scanned from −10° to +10° with a 1° increment. Intensity patterns on a detector are simple projections of the objects along certain angles according to the Radon transform as a forward model.

Filtered Backprojection (FBP)^3^ is chosen to perform backward operation. Here a crude estimate of *n* projections, i.e. ${\boldsymbol{g}}_1, \ldots ,\,{\boldsymbol{g}}_n$, using to the FBP algorithm without any regularization is the *n*th FBP Approximant or ${\boldsymbol{f}}_n^\prime$. Thus, the quality of the FBP Approximant is improved as *n* increases. As *n* spans from 1 to *N*(=21), a sequence of the FBP Approximants $\left( {{\boldsymbol{f}}_1^\prime ,\,{\boldsymbol{f}}_2^\prime ,\, \ldots ,\,{\boldsymbol{f}}_N^\prime } \right)$ becomes the input to an encoder and a recurrence cell as shown in Fig. 1b. The FBP Approximant sequences for training, validation, and testing are generated with these procedures and based on three-dimensional simulated phantoms.

However, under the strong scattering condition, the dense-layered, binary-phase object is illuminated at a sequence of angles, and the corresponding diffraction intensity patterns are captured by a detector. At the *n*th step of the sequence, the object is illuminated by a plane wave at angles $\left( {\theta _{nx},\,\theta _{ny}} \right)$ with respect to the propagation axis *z* on the *xz* and *yz* planes, respectively. Beyond the object, the scattered field propagates in free space by distance Δ*z* to the digital camera (the numerical value is Δ*z* = 58.2 mm. Let the forward model under the *n*th illumination angle be denoted as $H_n,\,n = 1,2, \ldots ,\,N$; that is, the *n*th measurement at the detector plane produced by the phase object ***f*** is ***g***_*n*_.

The forward operators *H*_*n*_ are obtained from the non-paraxial BPM^33,40,45^, which is less usual so we describe it in some additional detail here. Let the *j*th cross-section of the computational window perpendicular to *z*-axis be $f^{\left[ j \right]} = \exp \left( {i\varphi ^{\left[ j \right]}} \right),\,j = 1, \ldots ,\,J,$ where *J* is the number of slices the we divide the object into, each of axial extent *δz*. The optical field at the (*j* + 1)th slice is expressed as

1 $$\psi _n^{\left[ {j + 1} \right]} = {\cal{F}}^{ - 1}\left[ {{\cal{F}}\left[ {\psi _n^{[j]} \circ f_n^{\left[ j \right]}} \right]\left( {k_x,k_y} \right) \cdot \exp \left( { - i\left( {k - \sqrt {k^2 - k_x^2 - k_y^2} } \right)\delta z} \right)} \right]$$

where *δz* is is equal to the slab thickness, i.e. 0.5 mm; ${\cal{F}}$ and ${\cal{F}}^{ - 1}$ are the Fourier and inverse Fourier transforms, respectively; and $\chi _1 \circ \chi _2$ denotes the Hadamard (element-wise) product of the functions $\chi _1$ and $\chi _2.$The Hadamard product is the numerical implementation of the thin transparency approximation, which is inherent in the BPM. To obtain the intensity at the detector, we define the (*J* + 1)th slice displaced by Δ*z* from the *J*th slice (the latter is the exit surface of the object) to yield

2 $$H_n\left( {\boldsymbol{f}} \right) = \left| {\psi _n^{\left[ {J + 1} \right]}} \right|^2$$

The purpose of the Approximant, in general, is to produce a crude estimate of the volumetric reconstruction using the forward operator alone. This has been well established as a helpful form of pre-processing for subsequent treatment by machine learning algorithms^45,78^. Previous works constructed the Approximant as a single-pass gradient descent algorithm^33,45^. Here, due to the sequential nature of our reconstruction algorithm, as each intensity diffraction pattern from a new angle of illumination *n* is received, we instead construct a sequence of Approximants, indexed by *n*, by solving the problem

3 $$\begin{array}{*{20}{l}}{\hat{\boldsymbol f}} = {\mathrm{argmin}}_{\boldsymbol{f}}{\cal{L}}_{n} \left( {\boldsymbol{f}} \right)\,{\mathrm{where}} \, {\cal{L}}_{n} \left( {\boldsymbol{f}} \right) = \displaystyle\frac{1}{2} \Vert {H_n} \left( {\boldsymbol{f}} \right) - {\boldsymbol{g}}_{n} \Vert_{2}^{2}, \\ n= 1,2, \ldots, \, N \end{array}$$

The gradient descent update rule for this functional ${\cal{L}}_n\left( {\boldsymbol{f}} \right)$ is

4 $${\boldsymbol{f}}_n^{\left[ {l + 1} \right]} = {\boldsymbol{f}}_n^{\left[ l \right]} - s\left( {\nabla _{\boldsymbol{f}}{\cal{L}}_n\left( {{\boldsymbol{f}}_n^{\left[ l \right]}} \right)} \right)^\dagger = {\boldsymbol{f}}_n^{\left[ l \right]} - s\left( {H_n^T\left( {{\boldsymbol{f}}_n^{\left[ l \right]}} \right)\nabla _{\boldsymbol{f}}H_n\left( {{\boldsymbol{f}}_n^{\left[ l \right]}} \right) - {\boldsymbol{g}}_n^T\nabla _{\boldsymbol{f}}H_n\left( {{\boldsymbol{f}}_n^{\left[ l \right]}} \right)} \right)^\dagger$$

where ${\boldsymbol{f}}_n^{\left[ 0 \right]} = 0$ and *s* is the descent step size and in the numerical calculations was set to 0.05 and the superscript † denotes the transpose. The single-pass, gradient descent-based Approximant was used for training and testing of the RNN with simulated diffraction patterns but with an additional pre-processing step that will be explained in Eq. (7).

We also implemented a denoised TV-based Approximant, to be used only at the testing stage of the RNN with experimental diffraction patterns, where the additional pre-processing step in Eq. (7) also applies. In this case, the functional to be minimized is

5 $$\begin{array}{*{20}{l}}{\cal{L}}_n^{{\mathrm{TV}}}\left( {\boldsymbol{f}} \right) = \displaystyle\frac{1}{2} \Vert H_n\left( {\boldsymbol{f}} \right) - {\boldsymbol{g}}_{n} \Vert_2^2 + \kappa {\mathrm{TV}}_{l_1}\left( {\boldsymbol{f}} \right), \\\quad\quad\;\;\; n = 1,2, \ldots ,\,N \end{array}$$

where the TV-regularization parameter was chosen as $\kappa = 10^{ - 3}$, and for ${\boldsymbol{x}} \in {\cal{R}}^{P \times Q}$ the anisotropic *l*_1_-TV operator is

6 $${\mathrm{TV}}_{l_1}\left( {\boldsymbol{x}} \right) = \mathop {\sum }\limits_{p = 1}^{P - 1} \mathop {\sum }\limits_{q = 1}^{Q - 1} \left( {\left| {x_{p,q} - x_{p + 1,q}} \right| + \left| {x_{p,q} - x_{p,q + 1}} \right|} \right) + \mathop {\sum }\limits_{p = 1}^{P - 1} \left| {x_{p,Q} - x_{p + 1,Q}} \right| + \mathop {\sum }\limits_{q = 1}^{Q - 1} \left| {x_{P,q} - x_{P,q + 1}} \right|$$

with reflexive boundary conditions^79,80^. To produce the Approximants of experimentally obtained diffraction patterns for testing from this functional, we first ran 4 iterations of the gradient descent and ran 8 iterations of the FGP-FISTA (Fast Gradient Projection with Fast Iterative Shrinkage Thresholding Algorithm)^79,81^.

### Dynamically weighted moving average

The *N* Approximants of the strong scattering case form a 4D spatiotemporal sequence $\left( {{\boldsymbol{f}}_1^{\left[ 1 \right]},{\boldsymbol{f}}_2^{[1]}, \ldots ,\,{\boldsymbol{f}}_N^{[1]}} \right),$ which we process with a Dynamical Weighted Moving Average (DWMA) operation. For the weak scattering case, we omit this operation. The purpose of the DWMA is to smooth out short-term fluctuations, such as experimental artifacts in raw intensity measurements, and highlight longer-term trends, e.g. the change of information conveyed by different forward operators along the angular axis. The resulting DWMA Approximants ${\tilde{\boldsymbol {f}}}_m^{[1]}$ have a shorter length *M* than the original Approximants ${\boldsymbol{f}}_n^{[1]}$, i.e. *M* < *N*. Also, the weights in the moving average are dynamically determined as follows.

7 $${\tilde{\boldsymbol {f}}}_m^{[1]} = \left\{ {\begin{array}{*{20}{l}} \sum \limits_{n = m}^{m + N_w} \alpha _{nm}{\boldsymbol{f}}_n^{[1]}, \hfill & {1 \le m \le N_h} \hfill \\ \sum\limits_{n = m}^{m + N_w} \alpha _{nm}{\boldsymbol{f}}_{n + N_w}^{[1]}, \hfill & {N_h + 1 \le m \le M} \hfill \end{array}} \right.$$

where $$e_{nm} = \tanh \left( W_e^mf_n^{\left[1\right]} \right)$$

$$\begin{array}{*{20}{l}}\alpha _{nm} = {\mathrm{softmax}}\left( {e_{nm}} \right) = \frac{{\exp \left( {e_{nm}} \right)}}{\sum _{n = 1}^{N_w} \exp \left( {e_{nm}} \right)},\\ n = m,m + 1, \ldots ,m + N_w\end{array}$$

Equation 7 follows the convention of an additive attention mechanism^74^. *α*_*nm*_ indicates relative importance of ${\boldsymbol{f}}_n^{[1]}$ with respect to ${\tilde{\boldsymbol f}}_m^{[1]}$. Here, $W_e^m$ is a hidden layer assigned for each ${\tilde{\boldsymbol f}}_m^{[1]}$, which is subject to be trained for several epochs. The relative importance is determined by computing its associated energy *e*_*nm*_ and the softmax function normalizes it. More details are available in the Angular attention mechanism in Materials and methods. Supplementary Information (SI) Section S3 explains why the DWMA is more favorable than the Simple Moving Average (SMA) with fixed and uniform weights, i.e. 1/*M*.

To be consistent, the DWMA was applied to the original Approximants for both training and testing. In this study, *N*_*w*_ = 15, *N*_*h*_ = 6, *M* = 12. These choices follow from the following considerations. We have *N* = 42 diffraction patterns for each sequence: 21 captured along the *x*-axis (1 – 21; $\theta _x = - 10^\circ , - 9^\circ , \ldots ,\, + 10^ \circ$) and the remaining ones along the *y*-axis (22 – 42; $\theta _y = - 10^\circ , - 9^\circ , \ldots ,\, + 10^\circ$). The DWMA is first applied to 21 patterns from *x*-axis rotation, which thus generates 6 averaged diffraction patterns, and then it is applied to the remaining 21 patterns from *y*-axis rotation, resulting in the other 6 patterns. Therefore, the input sequence to the next step in the architecture of Fig. 16c, i.e. to the encoder (see Convolutional encoder and decoder in “Materials and methods” for details), consists of a sequence of *M* = 12 DWMA Approximants ${\tilde{\boldsymbol f}}_m^{[1]}.$ In SI Section S4, we discuss performance change due to different ways of numbering DWMA Approximants ${\tilde{\boldsymbol f}}_m^{[1]}$ entering the neural network. SI Section S5 provides visualization of DWMA Approximants.

### Separable-Convolution Gated Recurrent Unit (SC-GRU)

Recurrent neural networks involve a recurrent unit that retains memory and context based on previous inputs in a form of latent tensors or hidden units. It is well known that the LSTM is robust to instabilities in the training process. Moreover, in the LSTM, the weights applied to past inputs are updated according to usefulness, while less useful past inputs are forgotten. This encourages the most salient aspects of the input sequence to influence the output sequence^56^. Recently, the GRU was proposed as an alternative to LSTM. The GRU effectively reduces the number of parameters by merging some operations inside the LSTM, without compromising the quality of reconstructions; thus, it is expected to generalize better in many cases^69^. For this reason, we chose to utilize the GRU in this paper as well.

The governing equations of the standard GRU are as follows:

8 $$\begin{array}{*{20}{l}} {r_m} \hfill & = \hfill & \sigma({W_r\xi _m + U_rh_{m - 1} + b_r}) \hfill \\ {z_m} \hfill & = \hfill & \sigma({W_z\xi _m + U_zh_{m - 1} + b_z}) \hfill \\ {\tilde h_m} \hfill & = \hfill & {\tanh \left( {W\xi _m + U\left( {r_m \circ h_{m - 1}} \right) + b_h} \right)} \hfill \\ {h_m} \hfill & = \hfill & {\left( {1 - z_m} \right) \circ \tilde h_m + z_m \circ h_{m - 1}} \hfill \end{array}$$

where $\xi _m,\,h_m,\,r_m,\,z_m$ are the inputs, hidden features, reset states, and update states, respectively. Multiplication operations with weight matrices are performed in a fully connected fashion.

**Fig. 17:**
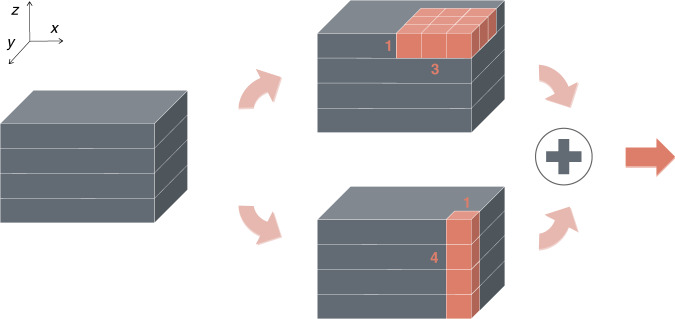
Separable-convolution scheme: different convolution kernels are applied along the lateral *x*, *y* axes vs. the longitudinal *z*-axis. In our present implementation, the kernels’ respective dimensions are 3 × 3 × 1 (or 1 × 1 × 1) and 1 × 1 × 4. The lateral and longitudinal convolutions are computed separately and the results are then added element-wise. The separable convolution scheme is used in both the gated recurrent unit and the encoder/decoder (for more details, see Convolutional encoder and decoder in Materials and methods)

We modified this architecture so as to take into account the asymmetry between the lateral and axial dimensions of optical field propagation, motivated from the concept of separable convolution in deep learning^82,83^ as shown in Fig. 17. This is evident even in free-space propagation, where the lateral components of the Fresnel kernel

9 $$\exp \left( {{\rm{i}}\pi \frac{{x^2 + y^2}}{{\lambda z}}} \right)$$

are shift invariant and, thus, convolutional, whereas the longitudinal axis *z* is not. The asymmetry is also evident in nonlinear propagation, as in the BPM forward model Eq. (1) that we used here. This does not mean that space is anisotropic – of course space is isotropic! The asymmetry arises because propagation and the object are 3D, whereas the sensor is 2D. In other words, the orientation of the image plane breaks the symmetry in object space so that the scattered field from a certain voxel within the object *apparently* influences the scattered intensity from its neighbors at the detector plane differently in the lateral direction than in the axial direction. To account for this asymmetry in a profitable way for our learning task, we first define the operators $W_r,\,U_r,$ etc. as convolutional so as to keep the number of parameters down (even though in free space propagation the axial dimension is not convolutional and under strong scattering neither dimension is nonlinear); and we constrain the convolutional kernels of the operators to be the same in the lateral dimensions *x* and *y*, and allow the axial *z* dimension kernel to be different. This approach justifies the term *separable convolution*, and we found it to be a good compromise between facilitating generalization and adhering to the physics of the problem.

We also replaced the tanh activation function of the standard GRU with ReLU activation^84^ as the ReLU is computationally less expensive and helpful to avoid local minima with fewer vanishing gradient problems^72,85^. The final form of our SC-GRU dynamics is

10 $$\begin{array}{*{20}{l}} {r_m} \hfill & = \hfill & \sigma({W_r \ast \xi _m + U_r \ast h_{m - 1} + b_r}) \hfill \\ {z_m} \hfill & = \hfill & \sigma({W_z \ast \xi _m + U_z \ast h_{m - 1} + b_z}) \hfill \\ {\tilde h_m} \hfill & = \hfill & {{\mathrm{ReLU}}\left( {W \ast \xi _m + U \ast \left( {r_m \circ h_{m - 1}} \right) + b_h} \right)} \hfill \\ {h_m} \hfill & = \hfill & {\left( {1 - z_m} \right) \circ \tilde h_m + z_m \circ h_{m - 1}} \hfill \end{array}$$

where * denotes the separable convolution operation.

### Convolutional encoder and decoder

CNNs are placed before and after the SC-GRU as encoder and decoder, respectively. This architectural choice was inspired by refs. ^86–89^. The encoder and decoder also utilize separable convolution, in conjunction with residual learning, which is known to improve generalization in deep networks^90^. As in ref. ^86^, the encoder and decoder utilize Down-Residual Blocks (DRB), Up-Residual Blocks (URB), and Residual Blocks (RB), whose details can be found in Fig. S5 in SI Section S6; however, there are no skip connections in our case, i.e. this is not a U-net^91^ architecture. The encoder learns how to map its input (i.e. the FBP Approximant ${\boldsymbol{f}}_n^\prime$ or the DWMA Approximant ${\tilde{\boldsymbol f}}_m^{[1]}$ sequence) onto a low-dimensional nonlinear manifold. For the weak scattering case, the compression factor for both lateral and axial input dimensions is 8, whereas for the strong scattering case, the compression factor is 16 for the lateral input dimensions, but the axial dimension is left intact. This eases the burden on the training process as the number of parameters is reduced; more importantly, encoding abstracts features out of the high-dimensional inputs, passing latent tensors over to the recurrent unit. Letting the encoder for the *m*th Approximant be symbolized as ${\mathrm{Enc}}_m\left( \cdot \right)$, $\xi _m = {\mathrm{Enc}}_m\left( {{\boldsymbol{f}}_m^\prime } \right)$ or $\xi _m = {\mathrm{Enc}}_m\left( {{\tilde{\boldsymbol f}}_m^{\left[ 1 \right]}} \right)$ in Eq. (10). The decoder restores the output of the RNN to the native dimension of the object we are reconstructing.

### Angular attention mechanism

Each raw image (either a projection under a weak scattering assumption or a diffraction pattern under a strong scattering condition) from a new angle of illumination is combined at the SC-GRU input with the hidden feature from the same SC-GRU’s previous output. After *N* iterations, there are *N* different hidden features resulting from *N* illumination angles, as seen in Eq. (10). Since the forward operator under both scattering assumptions is object dependent, the qualitative information that each such new angle conveys will vary with the object. It then becomes interesting to consider whether some angles of illumination convey more information than others.

The analog in temporal dynamical systems, the usual domain of application for RNNs, is the *attention* mechanism. It decides which elements of the system’s state are the most informative. In our case, of course, time has been replaced by the angle of illumination, so we refer to the same mechanism as *angular attention*: it evaluates the relative importance of information from each illumination angle in generating the overall reconstruction and thus adaptively assigns different weights to every angle on how much attention should be paid to.

Following the convention of an additive attention mechanism^74^, we compute the weight *α*_*m*_ from its associated energy *e*_*m*_ as output of a neural network with a hidden layer *W*_*e*_ as

11 $$\begin{array}{*{20}{l}} e_m = \tanh \left( {W_eh_m} \right), \\ \alpha _m = {\mathrm{softmax}}\left( {e_m} \right) = \frac{{\exp \left( {e_m} \right)}}{{\mathop {\sum }\nolimits_{m = 1}^M \exp \left( {e_m} \right)}},\\ m = 1,2, \ldots ,\,M \end{array}$$

The output of the angular attention as a single representation *a* is then computed from a linear combination of the hidden features as

12 $$a = \mathop {\sum }\limits_{m = 1}^M \alpha _mh_m$$

where *a* can be also viewed as the expected hidden representation since the weight *α*_*m*_ is essentially a probability that a hidden representation *h*_*m*_ is taken into account to the angular attention output *a*. There is an alternative, dot-product attention mechanism^92^, but we chose not to implement it here.

### Training the recurrent neural network

For the weak scattering case, 2000 and 400 synthetic amplitude phantoms are used for training and validation, respectively. Projections are acquired by the Radon transform along several angles within the limited angular range, as described in Approximant computations in Materials and methods. The FBP Approximants are obtained by the FBP algorithm from the projections.

For the strong scattering case, 5000 and 500 synthetic layered objects are used for training and validation, respectively. For each object, a sequence of intensity diffraction patterns from the *N* = 42 angles of illumination are produced by BPM, as described earlier. The Approximants are obtained each as a single iteration of the gradient descent, followed by the DWMA process.

For both scattering cases, all of the architectures are trained with a Training Loss Function (TLF) of negative Pearson Correlation Coefficient (NPCC)^93^:

13 $${\cal{E}}_{{\mathrm{NPCC}}}\left( {f,\hat f} \right) = - \frac{{\mathop {\sum }\nolimits_{x,y} \left( {f\left( {x,y} \right) - f} \right)\left( {\hat f\left( {x,y} \right) - \hat f} \right)}}{{\sqrt {\mathop {\sum }\nolimits_{x,y} \left( {f\left( {x,y} \right) - f} \right)^2} \sqrt {\mathop {\sum }\nolimits_{x,y} \left( {\hat f\left( {x,y} \right) - \hat f} \right)^2} }}$$

where *f* and $\hat f$ are a ground truth image and its corresponding reconstruction. In this article, our NPCC function is defined to perform computation in 3D. We use a stochastic gradient descent scheme with the *Adam* optimizer^94^. The learning rate is set to be 10^−3^ initially and halved whenever validation loss plateaued for 5 consecutive epochs. The lower bound is set to be 10^−6^, and the batch size is set to be 10. The computer used for training has Intel Xeon Gold 6248 CPU at 2.50 GHz with 27.5 MB cache, 384 GB RAM, and dual NVIDIA Volta V100 GPUs with 32 GB VRAM^95^, and it took approximately 5 min per each training epoch.

For comparison, we also re-train the 3D-DenseNet architecture with skip connections in ref. ^45^ with the same training scheme above. This serves as baseline; however, the number of parameters in this network is 0.5 M, whereas in our RNN architecture the number of parameters is 21 M. We also train an enhanced version of the 3D-DenseNet by tuning the number of dense blocks, the number of layers inside each dense block, filter size, and growth rate to match the total number of parameters with that of the RNN, i.e. 21 M. These two versions of the 3D-DenseNet are referred to as Baseline (0.5 M) and Baseline (21 M), respectively, in Figs. 8, 9, 11, and 12.

### Testing procedures and metrics

Test performance was demonstrated with only the simulated projections under the weak scattering condition. The projections were processed with the FBP to generate 100 sequences of FBP Approximants ${\boldsymbol{f}}_n^\prime$ for testing the trained network. Under the strong scattering condition, both simulated and experimental diffraction patterns were used for testing, but the patterns were processed differently. The simulated patterns were directly processed with a single-pass gradient descent to generate 50 sequences of original Approximants ${\boldsymbol{f}}_n^{\left[ 1 \right]}$ Eq. (4), whereas a simple affine transform was first applied to the raw experimentally obtained intensity diffraction patterns of an actual layered object to correct slight misalignment. We then applied the gradient descent up to 4 iterations and the FGP-FISTA up to 8 iterations to the corrected experimental patterns, to compute one set of TV-based Approximants Eq. (5). Testing process took approximately 300 ms for generating each volumetric reconstruction.

Even though training used NPCC as in Eq. (13). we investigated two additional metrics for testing: PE and the Wasserstein distance^96,97^. We also quantified test performance using the SSIM^98^.

PE is the mean absolute error between two binary objects; in the digital communication community it is instead referred to as Bit Error Rate (BER). It is the first time to our knowledge to use PE as a quantitative metric in tomography. To obtain the PE, we first threshold the reconstructions (see SI Section S7 for details in the thresholding process) and then define

14 $${\mathrm{PE = }}\frac{{\left( {\# \,{\mathrm{false}}\,{\mathrm{negatives}}} \right) + \left( {\# \,{\mathrm{false}}\,{\mathrm{positives}}} \right)}}{{{\mathrm{total}}\,\# \,{\mathrm{pixels}}}}$$

We found that it oftentimes helps to accentuate the differences between a binary phase ground truth object and its binarized reconstruction as even small residual artifacts, if they are above the threshold, are thresholded to be one, and thus they are taken into account to the probability of error calculation more than they would have been to other metrics. With these procedures, PE is a particularly suitable error metric for the kind of objects we consider in this paper.

PE is also closely related to the two-dimensional Wasserstein distance as we will now show through an analytical derivation. The latter metric involves an optimization process in terms of a transport plan to minimize the total cost of transport from a source distribution to a target distribution. The two-dimensional Wasserstein distance is defined as

15 $$\begin{array}{*{20}{l}} {W_{p = 1}} \hfill & = \hfill & {\mathop {{{\mathrm{min}}}}\limits_p \,{\it{P,C}}} \hfill & = \hfill & {\mathop {{{\mathrm{min}}}}\limits_p \,\mathop {\sum }\limits_{ij} \mathop {\sum }\limits_{kl} \gamma _{ij,kl}C_{ij,kl}} \hfill \\ {{\mathrm{s}}.{\mathrm{t}}{\mathrm{.}}\mathop {\sum }\limits_{kl} \gamma _{ij,kl}} \hfill & = \hfill & {f_{ij},\mathop {\sum }\limits_{ij} \gamma _{ij,kl}} \hfill & = \hfill & {g_{kl},\,\gamma _{ij,kl} \ge 0} \hfill \end{array}$$

where *f*_*ij*_ and *g*_*kl*_ are a ground truth binary object and its binary reconstruction, i.e. $f_{ij},\,g_{kl},\,\gamma _{ij,kl} \in \left\{ {0,1} \right\}$, a coupling tensor $P = \left( {\gamma _{ij,kl}} \right)$, and a cost tensor $C_{ij,kl} = \left| {x_{ij} - x_{kl}} \right|.$ PE can be reduced to have a similar, but not equivalent, form to that of the Wasserstein distance. For *i*.*j*,*k*,*l* where $\gamma _{ij,kl} \, \ne \, 0$,

16 $$\begin{array}{*{20}{l}} {{\mathrm{PE}}} \hfill & = \hfill & {\displaystyle \frac{1}{{N^2}}\mathop {\sum }\limits_{ij} \left| {f_{ij} - g_{ij}} \right|} \hfill \\ {\,} \hfill & = \hfill & {\displaystyle \frac{1}{{N^2}}\mathop {\sum }\limits_{ij} \left| {f_{ij} - \mathop {\sum }\limits_{kl} g_{kl}\delta \left[ {i - k,j - l} \right]} \right|} \hfill \\ {\,} \hfill & = \hfill & {\displaystyle \frac{1}{{N^2}}\mathop {\sum }\limits_{ij} \left| {\mathop {\sum }\limits_{kl} \gamma _{ij,kl} \displaystyle \left( {1 - \frac{{g_{kl}\delta \left[ {i - k,j - l} \right]}}{{\gamma _{ij,kl}}}} \right)} \right|} \hfill \\ {\,} \hfill & = \hfill & {\mathop {\sum }\limits_{ij} \left| {\mathop {\sum }\limits_{kl} \gamma _{ij,kl}\,\tilde C_{ij,kl}} \right|} \hfill \\ {\,} \hfill & = \hfill & {\mathop {\sum }\limits_{ij,kl,\gamma _{ij,kl} \ne 0} \gamma _{ij,kl}\,\tilde C_{ij,kl}} \hfill \end{array}$$

where $$N^2\,\tilde C_{ij,kl} = 1 - \frac{{g_{kl}\delta \left[ {i - k,j - l} \right]}}{{\gamma _{ij,kl}}} = \left\{ {\begin{array}{*{20}{l}} {1,} \hfill & {if\,ij \, \ne \, kl} \hfill \\ {1 - g_{kl}} \hfill & {if\,ij = kl} \hfill \end{array}} \right.$$

This shows that the PE is a version of the Wasserstein distance with differently defined cost tensor.

## Supplementary information

Supplementary information

Supplementary movie 1

Supplementary movie 2

Supplementary movie 3

Supplementary movie 4
