# Correction: Dynamical machine learning volumetric reconstruction of objects’ interiors from limited angular views

**DOI:** 10.1038/s41377-021-00615-5

**Published:** 2021-09-03

**Authors:** Iksung Kang, Alexandre Goy, George Barbastathis

**Affiliations:** 1 Department of Electrical Engineering and Computer Science, Massachusetts Institute of Technology, 77 Massachusetts Ave, Cambridge, MA USA; 2 Department of Mechanical Engineering, Massachusetts Institute of Technology, Cambridge, MA 02139 USA; 3 Singapore-MIT Alliance for Research and Technology (SMART) Centre, 1 Create Way, Singapore, 117543 Singapore; 4 Present Address: Omnisens SA, Morges, 1110 Switzerland

**Keywords:** Applied optics, Imaging and sensing

Correction to: *Light: Science & Applications*

10.1038/s41377-021-00512-x, published online 07 April 2021

Iksung Kang, Alexandre Goy, George Barbastathis

We are correcting Eq. (8) on p. 16 and Eq. (10) on p. 17, which were inadvertently missing sigmoid functions ($\sigma$).

Eq. (8) should be corrected as:

$$r_m = \sigma \left( {W_r\xi _m + U_rh_{m - 1} + b_r} \right)$$

$$z_m = \sigma \left( {W_z\xi _m + U_zh_{m - 1} + b_z} \right)$$

$$\tilde h_{{{\mathrm{m}}}} = \tanh \left( {W\xi _m + U\left( {r_m \circ h_{m - 1}} \right) + b_h} \right)$$

$$h_m = \left( {1 - z_m} \right) \circ \tilde h_m + z_m \circ h_{m - 1}$$

and Eq. (10) should be corrected as:

$$r_m = \sigma \left( {W_r \ast \xi _m + U_r \ast h_{m - 1} + b_r} \right)$$

$$z_m = \sigma \left( {W_z \ast \xi _m + U_z \ast h_{m - 1} + b_z} \right)$$

$$\tilde h_{{{\mathrm{m}}}} = {{{\mathrm{ReLU}}}}\left( {W \ast \xi _m + U \ast \left( {r_m \circ h_{m - 1}} \right) + b_h} \right)$$

$$h_m = \left( {1 - z_m} \right) \circ \tilde h_m + z_m \circ h_{m - 1}$$

The code is publicly available at https://github.com/iksungk.

The original article has been updated.

