# Supplementary material for: Dynamical machine learning volumetric reconstruction of objects’ interiors from limited angular views
Source: Light Sci Appl. 2021 Apr 7;10:74. doi: 10.1038/s41377-021-00512-x (PMC8027224; doi:10.1038/s41377-021-00512-x)
Supplement: Supplementary file 1 — Supplementary information [file 41377_2021_512_MOESM1_ESM.docx]

**Supplementary Information (SI) for**

**Dynamical machine learning volumetric reconstruction of objects’ interiors from limited angular views**

Iksung Kang^1*^, Alexandre Goy^2†^, George Barbastathis^2,3^

^1^ Department of Electrical Engineering and Computer Science, Massachusetts Institute of Technology, 77 Massachusetts Ave, Cambridge, MA 02139, USA.

^2^ Department of Mechanical Engineering, Massachusetts Institute of Technology, Cambridge, MA 02139, USA.

^3^ Singapore-MIT Alliance for Research and Technology (SMART) Centre, 1 Create Way, Singapore 117543, Singapore.

^†^ Present address: Omnisens SA, Morges 1110, Switzerland

^*^ Corresponding author: [iskang@mit.edu](mailto:iskang@mit.edu)

**S1. Details of parameters of the simulations used in Figures 8 and 9**

Table S1. Parameters of simulated phantoms used in Figures 8 and 9. Units are normalized, so the diameter and the center x and y coordinates of each phantom are within [0, 1] and [-1, 1], respectively.

|  | **Sparse** | **Dense** | **Small** | **Large** |
| --- | --- | --- | --- | --- |
| **Diameter** | [0.2, 0.4] | [0.2, 0.4] | [0.2, 0.5] | [0.5, 0.8] |
| **Center coordinates** | [-1, 1] | [-0.6, 0.6] | [-0.5, 0.5] | [-0.5, 0.5] |
| **Number of phantoms** | 3 | 8 | [1, 4] | [1, 4] |

**S2. Tabulation of some results in the main manuscript**

| **PCC** | Layer 1 | Layer 2 | Layer 3 | Layer 4 | Overall |
| --- | --- | --- | --- | --- | --- |
| Proposed RNN | **0.8580** | **0.6512** | **0.9539** | 0.6228 | **0.7715** |
| Baseline (0.5 M) | 0.8453 | 0.5058 | 0.8751 | 0.5558 | 0.6955 |
| Baseline (21 M) | 0.8487 | 0.5925 | 0.8917 | **0.6419** | 0.7437 |

| **SSIM** | Layer 1 | Layer 2 | Layer 3 | Layer 4 | Overall |
| --- | --- | --- | --- | --- | --- |
| Proposed RNN | **0.7449** | **0.7218** | **0.8767** | **0.7910** | **0.7836** |
| Baseline (0.5 M) | 0.6879 | 0.5566 | 0.7634 | 0.6310 | 0.6597 |
| Baseline (21 M) | 0.7123 | 0.6321 | 0.8044 | 0.7093 | 0.7145 |

| **Wasserstein distance (**$\boldsymbol{\times0.01}$**)** | Layer 1 | Layer 2 | Layer 3 | Layer 4 | Overall |
| --- | --- | --- | --- | --- | --- |
| Proposed RNN | **1.831** | **1.291** | **1.589** | **1.029** | **1.435** |
| Baseline (0.5 M) | 2.994 | 2.177 | 2.142 | 1.486 | 2.200 |
| Baseline (21 M) | 2.866 | 1.729 | 2.368 | 1.271 | 2.059 |

| **Probability of error (%)** | Layer 1 | Layer 2 | Layer 3 | Layer 4 | Overall |
| --- | --- | --- | --- | --- | --- |
| Proposed RNN | **6.580** | **4.431** | **2.393** | 1.910 | **3.828** |
| Baseline (0.5 M) | 7.233 | 5.969 | 6.677 | 2.399 | 5.569 |
| Baseline (21 M) | 6.934 | 5.011 | 5.762 | **1.831** | 4.884 |

**Table S2.** Tabulation of Figure 12b in the main manuscript.

| **PCC** | Layer 1 | Layer 2 | Layer 3 | Layer 4 | Overall |
| --- | --- | --- | --- | --- | --- |
| Proposed RNN | 0.8580 | 0.6512 | 0.9539 | 0.6228 | 0.7715 |
| (⇌) tanh | 0.8543 | 0.6647 | 0.9257 | 0.5962 | 0.7602 |
| (-) separable convolution | 0.8180 | 0.5752 | 0.7741 | 0.4894 | 0.6642 |
| (-) angular attention | **0.4171** | **0.4320** | **0.5996** | **0.3050** | **0.4384** |

| **SSIM** | Layer 1 | Layer 2 | Layer 3 | Layer 4 | Overall |
| --- | --- | --- | --- | --- | --- |
| Proposed RNN | 0.7449 | 0.7218 | 0.8767 | 0.7910 | 0.7836 |
| (⇌) tanh | 0.7389 | 0.7016 | 0.8338 | 0.7520 | 0.7566 |
| (-) separable convolution | 0.6909 | 0.6277 | 0.7036 | 0.7202 | 0.6856 |
| (-) angular attention | **0.3015** | **0.2631** | **0.4612** | **0.2362** | **0.3155** |

| **Wasserstein distance (**$\boldsymbol{\times0.01}$**)** | Layer 1 | Layer 2 | Layer 3 | Layer 4 | Overall |
| --- | --- | --- | --- | --- | --- |
| Proposed RNN | 1.831 | 1.291 | 1.589 | 1.029 | 1.435 |
| (⇌) tanh | 2.074 | 1.457 | 1.757 | 1.072 | 1.590 |
| (-) separable convolution | 2.837 | 1.498 | 5.139 | 1.363 | 2.709 |
| (-) angular attention | **7.705** | **4.401** | **6.219** | **3.813** | **5.543** |

| **Probability of error (%)** | Layer 1 | Layer 2 | Layer 3 | Layer 4 | Overall |
| --- | --- | --- | --- | --- | --- |
| Proposed RNN | 6.580 | 4.431 | 2.393 | 1.910 | 3.828 |
| (⇌) tanh | 6.665 | 4.211 | 3.845 | 2.087 | 4.202 |
| (-) separable convolution | 8.588 | 6.024 | 13.40 | 2.557 | 7.643 |
| (-) angular attention | **24.99** | **6.329** | **20.77** | **5.121** | **14.30** |

**Table S3.** Tabulation of Figure 14b in the main manuscript.

**S3. Comparison between the Dynamically Weighted Moving Average and the Simple Moving Average**


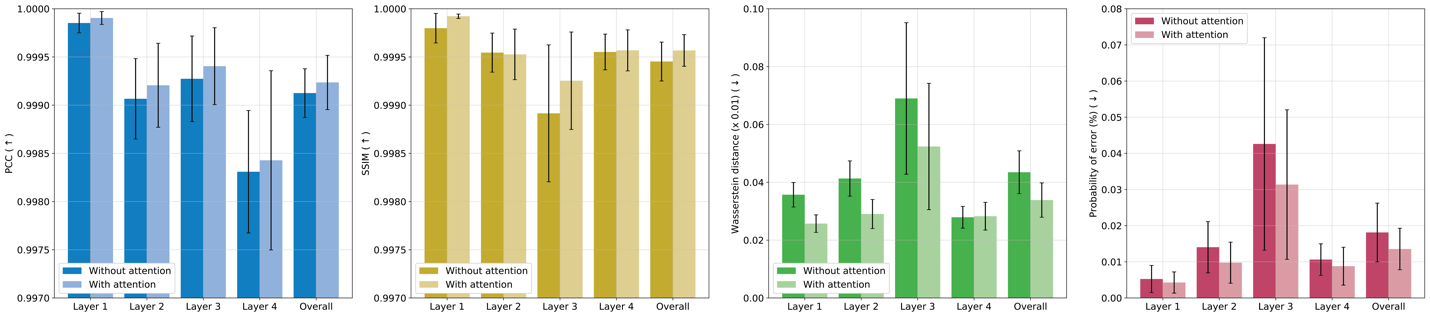


**Figure S1.** Four quantitative metrics (PCC, SSIM, Wasserstein distance, and Probability of error) show that the Dynamically Weighted Moving Average (DWMA) leads to better results. Simple Moving Average (SMA) corresponds to ‘without attention’, and DWMA to ‘with attention.’

Figure S1 is a supplementary figure to Eq. 7 in Materials and methods in the main manuscript. Eq. 7 illustrates the Dynamically Weighted Moving Average (DWMA) following the convention of the additive attention mechanism^1^, where each weight explains relative importance of its associated Approximant. Figure S1 compares the DWMA and the Simple Moving Average (SMA) with fixed and uniform weights.

**S4. Different ways of numbering Approximants entering the network**

Under the strong scattering condition, the network treats the DWMA Approximants ${\tilde{\boldsymbol{f}}}_{m}^{[1]}$ (see Eq. 7 in the main manuscript) with different weights as the training progresses thanks to the angular attention mechanism as it learns the weights from the training. For investigation on the attention mechanism, the DWMA Approximants are introduced to the network in three different sequences: normal, reversed, and randomized.

(S1)

$$\text{Normal sequence: }{\tilde{\boldsymbol{f}}}_{1}^{\left[ 1 \right]}, \ldots, {\tilde{\boldsymbol{f}}}_{6}^{\left[ 1 \right]},{\tilde{\boldsymbol{f}}}_{7}^{\left[ 1 \right]},\ldots, {\tilde{\boldsymbol{f}}}_{12}^{\left[ 1 \right]}$$

$$\text{Reversed sequence: }{\tilde{\boldsymbol{f}}}_{6}^{\left[ 1 \right]}, \ldots, {\tilde{\boldsymbol{f}}}_{1}^{\left[ 1 \right]},{\tilde{\boldsymbol{f}}}_{12}^{\left[ 1 \right]},\ldots, {\tilde{\boldsymbol{f}}}_{7}^{\left[ 1 \right]}$$

$$\text{Randomized sequence}: \text{12-permutations of }\left\{ m \right| 1,2,\ldots, 12\}$$

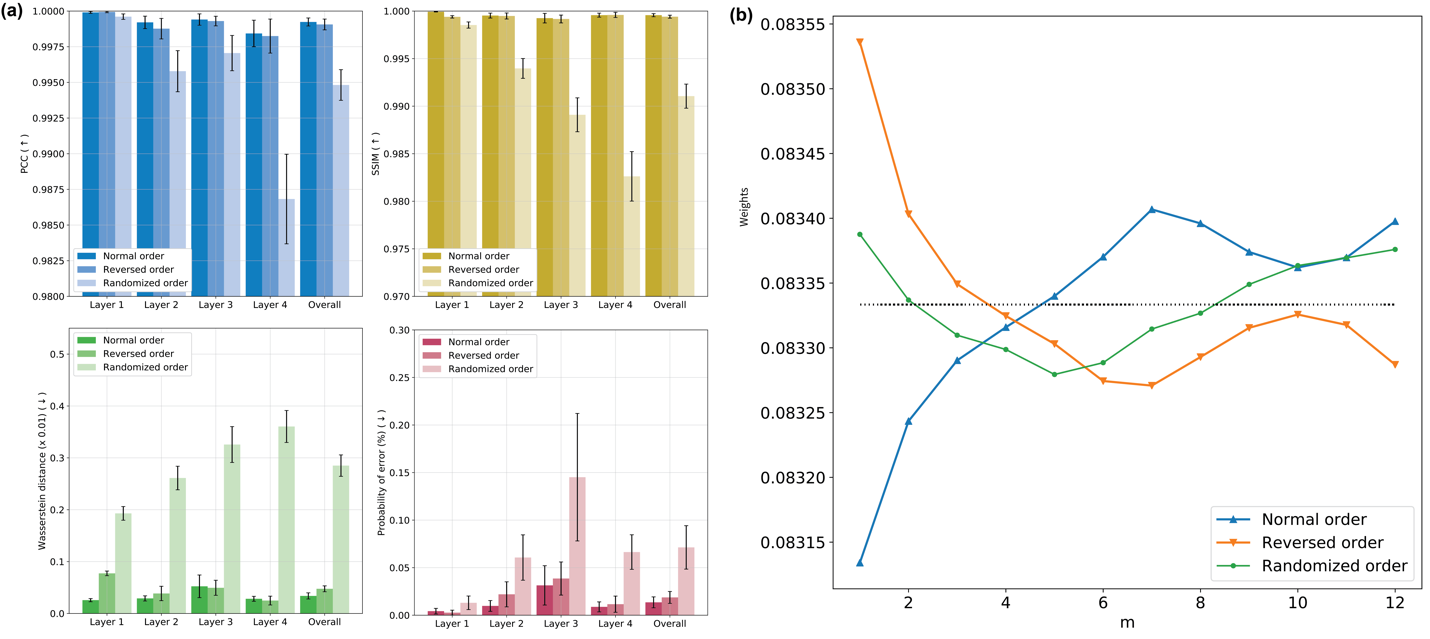


**Figure S2.** (a) PCC, SSIM, Wasserstein distance, and probability of error are computed on the four cases using simulated data. (b) Weights (or attention probabilities) of the angular attention mechanism on $h_{m}$ when the orders of Approximants are different in three ways.

Figure S2a agrees with our very first intuition that the results of the normal and reversed sequences should be more or less the same, and we know that the randomized sequence is proven to be deteriorating overall reconstruction quality. However, the networks trained with the Approximants in either the normal or reversed sequence have different trained weights of the angular attention mechanism.

As each hidden representation $h_{m}$ from the $m$-th recurrence is a nonlinear multivariate function of $\xi_{m}$ and $h_{m-1}$, *i.e.* $h_{m}=\sigma\left( h_{m-1}, \xi_{m} \right),$ and it also works recursively as $h_{m-1}$ is dependent on the previous hidden representations. Therefore, $h_{m}$ from the networks trained with either the normal or reversed order are no longer the same in general. (If $h_{m}$ were solely dependent on $\xi_{m}$, they could be trained to be the same.) For instance, although both the normal and reversed orders show less focus on  ${\tilde{\boldsymbol{f}}}_{1}^{[1]}$ and more focus on  ${\tilde{\boldsymbol{f}}}_{6}^{[1]}$, the actual trained weights associated with two Approximants differ.


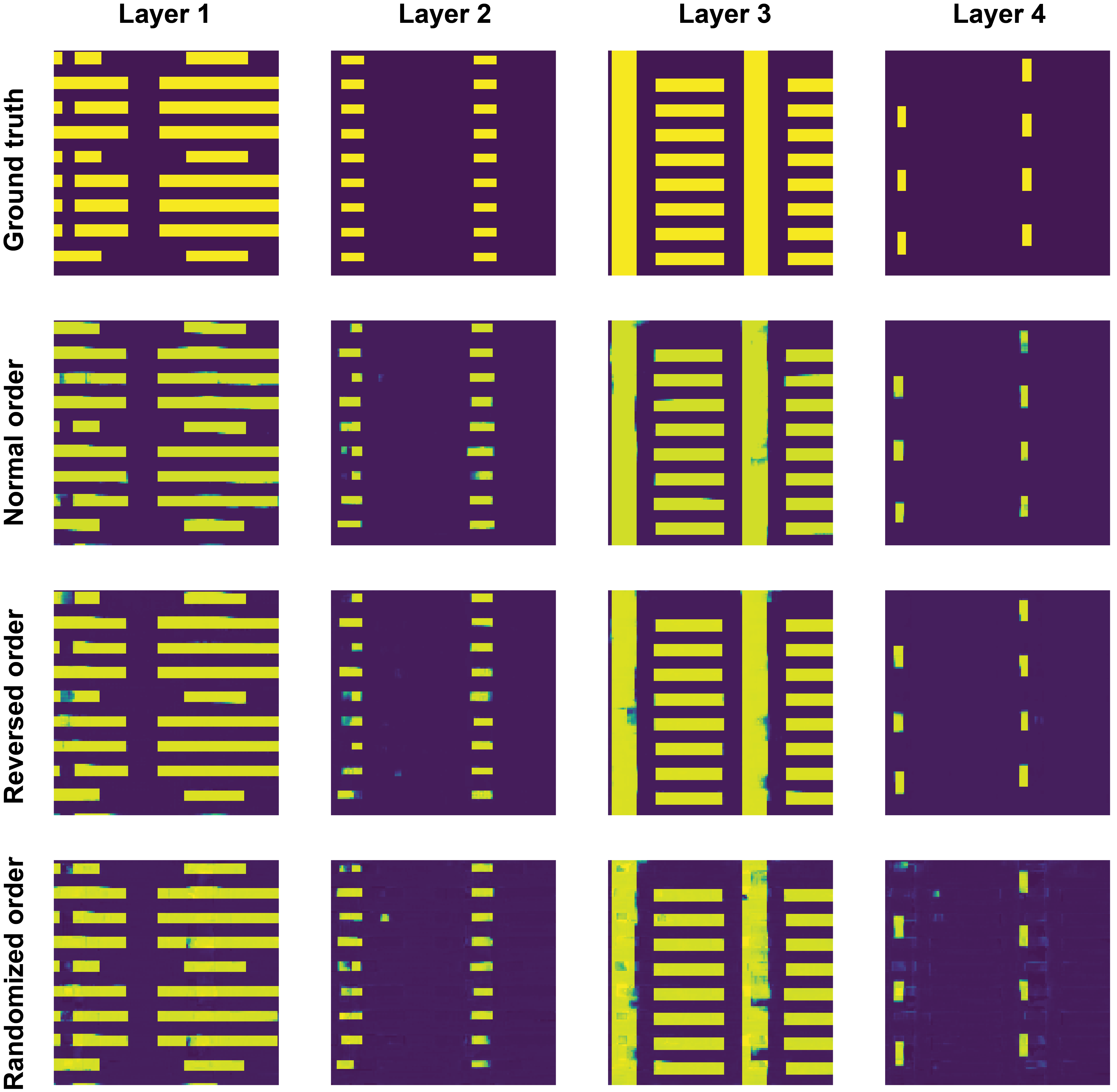


**Figure S3.** Qualitative comparison on reconstructions of when Approximants are entered to the network in different orders as suggested in Eq. S1.

In Figure S3, reconstructions from different orders of Approximants entering the network are qualitatively compared. These reconstructions are based on experimental measurements.

**S5. Examples of training and validation data for all datasets**

**
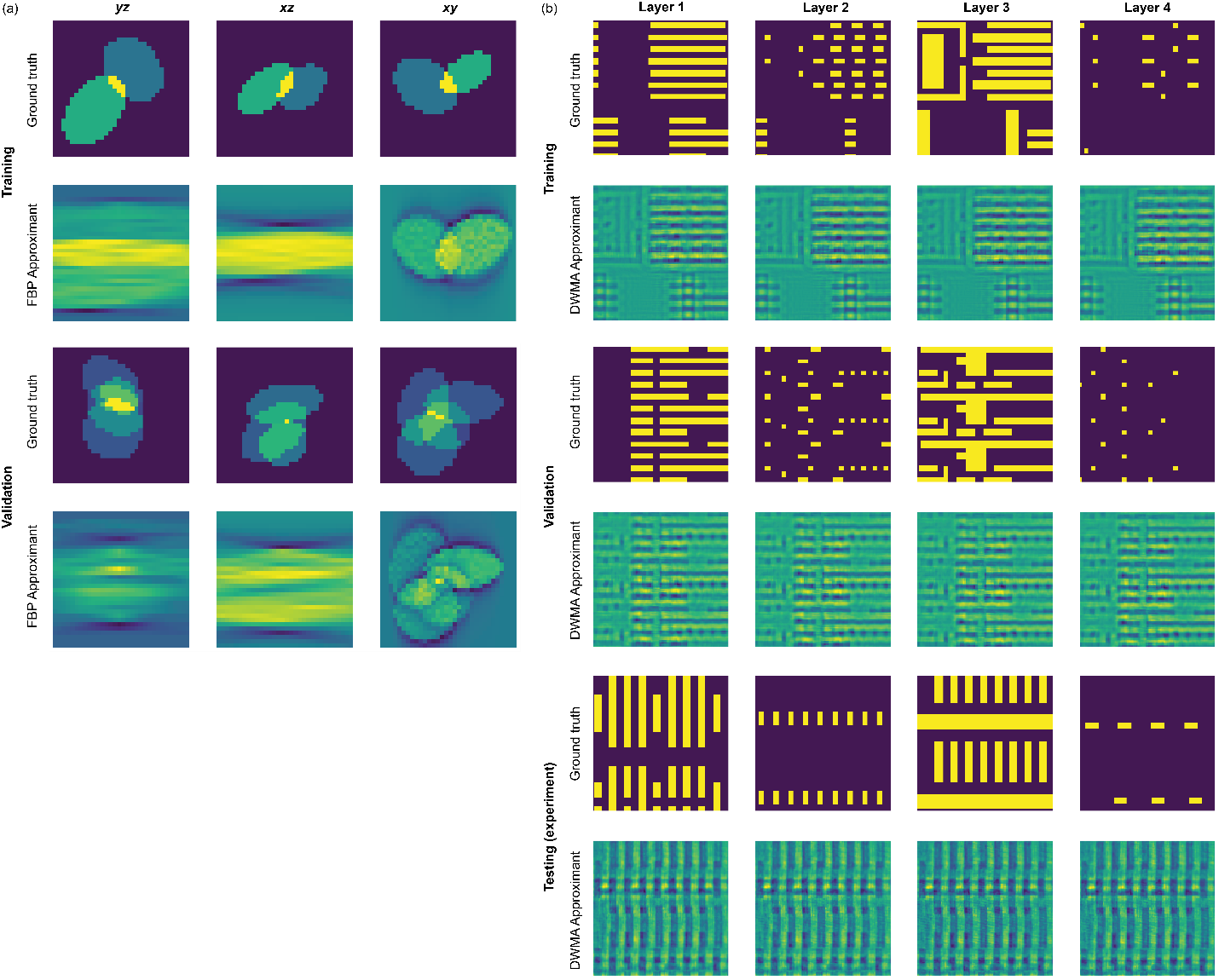
**

**Figure S4.** Input and output pairs of training and validation datasets of (a) the weak scattering and (b) strong scattering conditions. The testing pair in (b) is based on experimental measurements. Here, an FBP Approximant as an estimate of 21 projections and a DWMA Approximant ($m=12)$ are shown.

Figure S4 shows some examples of input and output pairs of training, validation, and testing datasets of both scattering conditions. For the weak scattering case, the input to the baseline model (21 M) is an FBP Approximant as an estimate of 21 projections, and the input to the proposed RNN (21 M) is a sequence of $N$ FBP Approximants, where each element is an estimate of $n$ projections ($n=1, \ldots, N (=21)$). For the strong scattering case, the input to the baseline models (0.5 M and 21 M) follow the conventions in ref. 4, and the input to the proposed RNN is a sequence of $M (=12)$ DWMA Approximants, which are defined in Materials and methods in the main manuscript.

**S6. Down-Residual Block (DRB), Up-Residual Block (URB), and Residual Block (RB)**


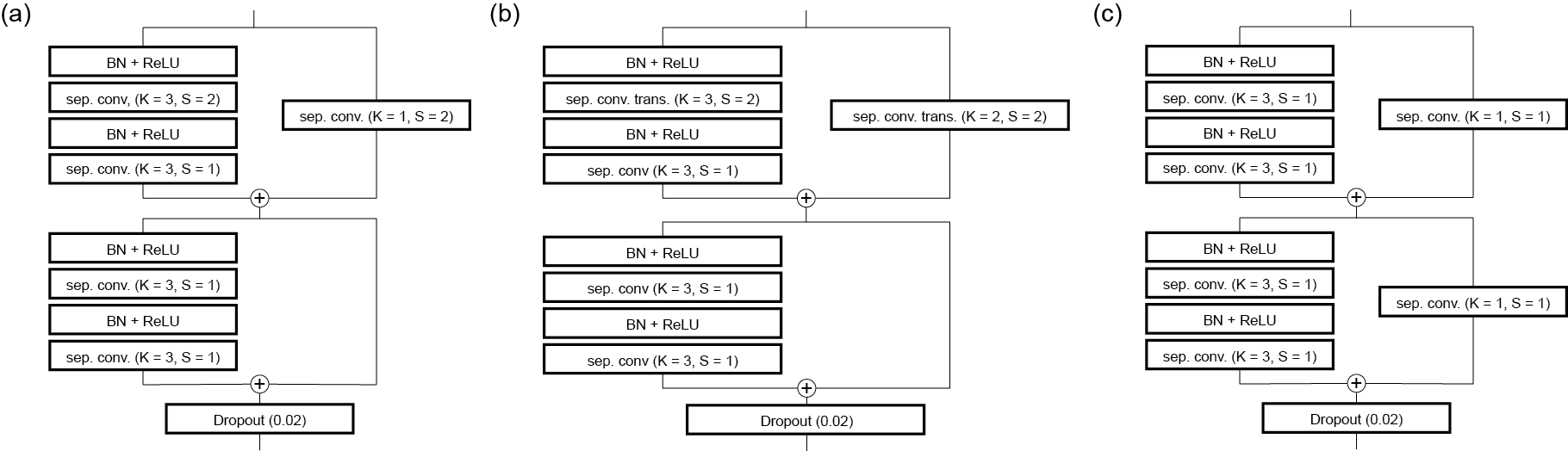


**Figure S5.** (a) Down-Residual Block (DRB), (b) Up-Residual Block (URB), and (c) Residual Block (RB). K and S indicate the sizes of kernel and stride, respectively, and the values shown apply only to the row and column axes. For the layer axis, K = 4 and S = 1 always. The disparities are to implement the separable convolution scheme; please see Separable-Convolution Gated Recurrent Unit (SC-GRU) in Methods.

**S7. Adaptive binarization process**


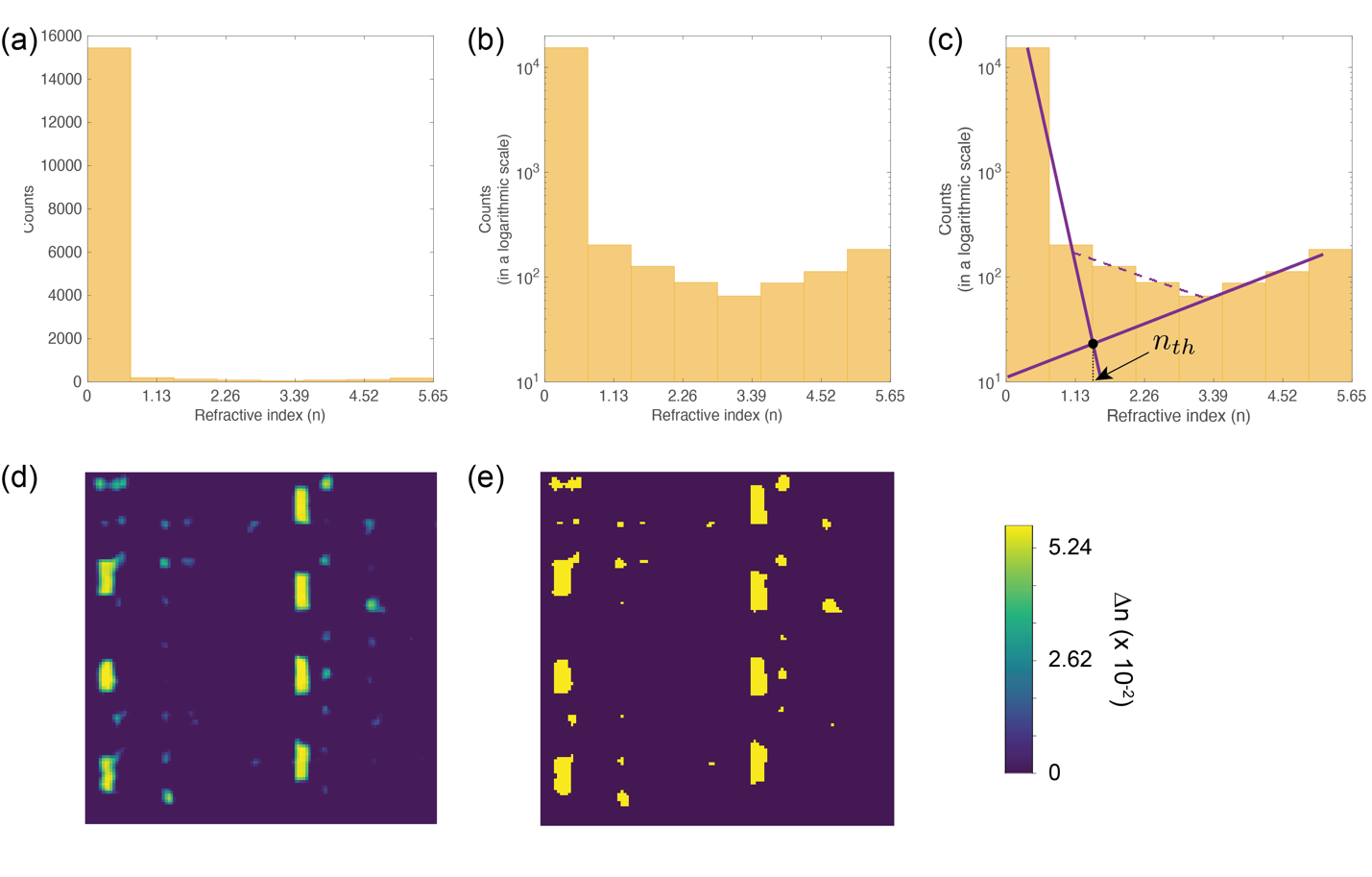


**Figure S6.** Adaptive binarization process. (a) Original histogram of (d) with the number of bins being 8. (b) The original histogram drawn in a logarthmic scale. (c) An overlay of (b) and its fitted piecewise-linear curve. According to the MAP decision rule, $n_{\text{th}}=1.4125$. (d) An example before binarization and (e) after binarization.

In this section, we explain how reconstructions are adaptively binarized. We adopt Maximum a Posteriori (MAP) decision rule in a logarithmic scale as shown in Figure S6, adaptively applied to each reconstruction to determine a threshold according to the decision rule. We bring this process to the logarithmic scale as histogram counts are mostly centered at the two binary values – we are interested in how to apply the threshold on residual artifacts with intermediate values that occupy only small regions in a reconstruction. We fit the histogram with a piecewise-linear curve consisting of three linear functions to apply the decision rule to separate the histogram into two distinct distributions. The first and the last linear functions represent the distributions on two binary values, and the middle one is barely the sum of the two. Thus, it is equivalent to fitting the original histogram with two one-sided exponential curves. According to the MAP decision rule, the threshold value $n_{\text{th}}$ is determined where the two linear functions meet. In Figure S6, $n_{\text{th}}=1.4125$*.*

Probability of error (PE), defined in Eq. 14 in the main manuscript, is computed on the binarized reconstructions as shown in Figures S7 and S8. PE is essentially the mean absolute error on the binarized reconstructions. This way, this metric may be an effective way to compute the error as even small residual artifacts are thresholded to be one if they are above the adaptively chosen threshold by the decision rule, and thus they are taken into consideration with a larger weight than they would have been to other metrics.


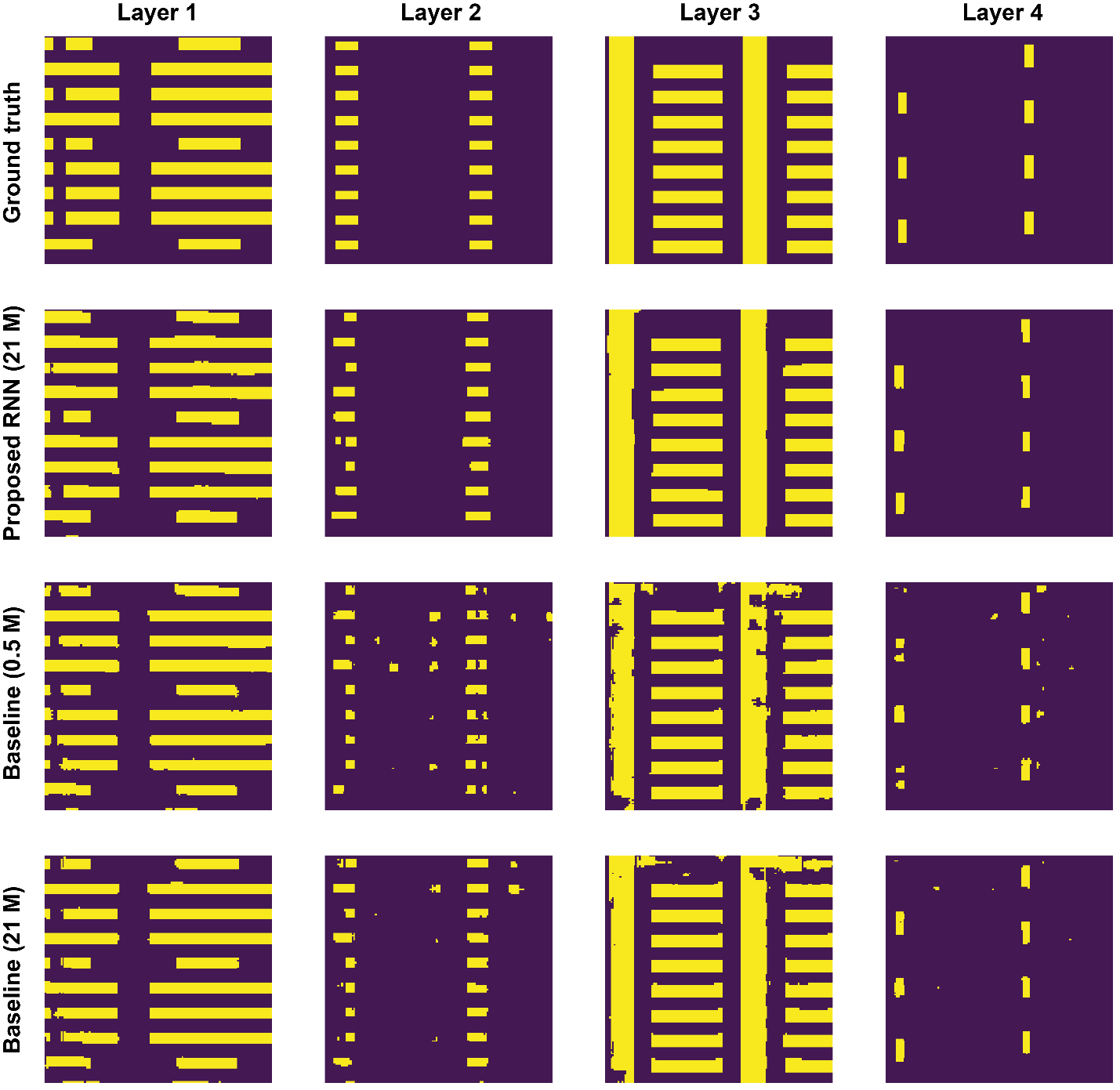


**Figure S7.** Binarized reconstructions of the baselines and proposed RNN. See the original reconstructions in Figure 11 in the main manuscript.


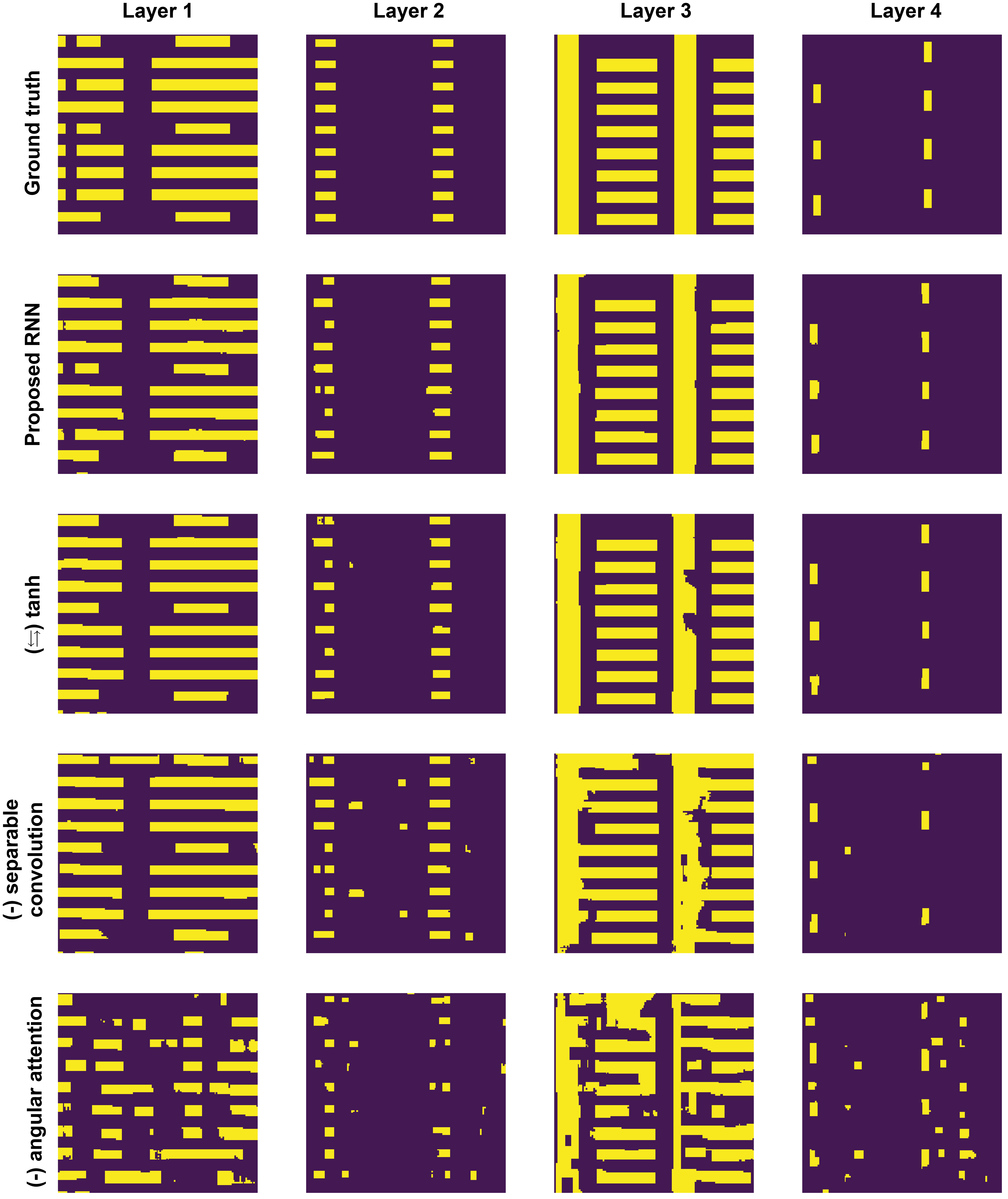


**Figure S8.** Binarized reconstructions of the ablation study. See the original reconstructions in Figure 13 in the main manuscript.

**S8. Performance of the RNN with different levels of noise**


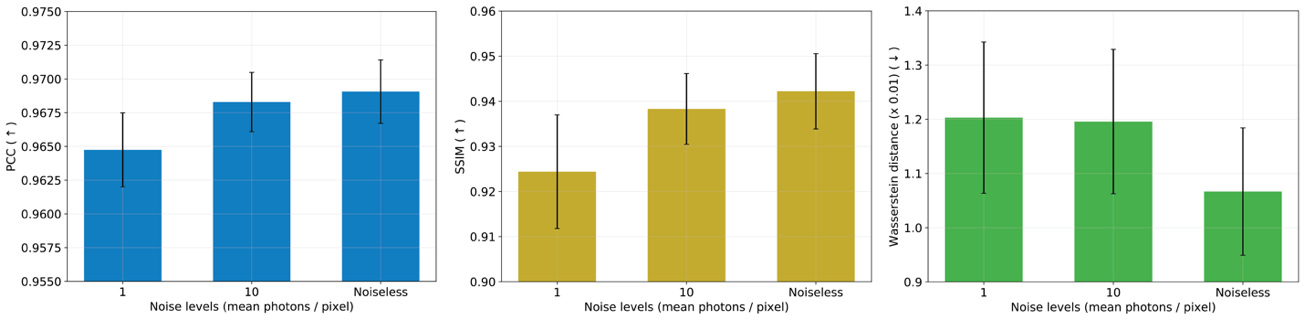


**Figure S9.** Quantitative comparison on performance of the RNN with different noise levels using three different metrics: PCC, SSIM, and Wasserstein distance. The graphs show means and 95% confidence intervals.

Using three different metrics, *i.e.* PCC, SSIM, and Wasserstein distance, we assess robustness of the network to noise. Here, we apply the noise to projections that follows purely Poisson to model photon noise under the weak scattering assumption. Figure S9 shows that all metrics degrade as the noise level increases, which is expected, but do not largely deviate from the noiseless case.

**S9. Scalability of the proposed RNN**

**
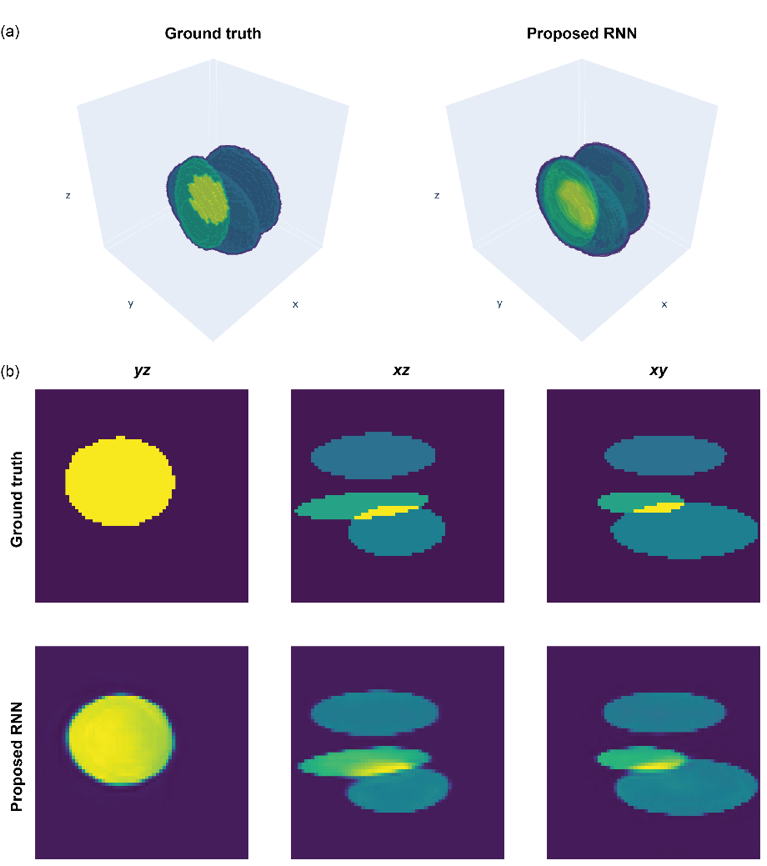
**

**Figure S10.** (a) 3D rendered volumes ($64\times64\times64$) of a reconstruction of the proposed RNN and its corresponding ground truth, and (b) 2D cross-section profiles.

Figure S10 shows 3D rendered volumes and 2D cross-section profiles of a reconstruction of the proposed RNN and its ground truth. Here, all volumes are $64\times64\times64$, which thus implies the scalability of the proposed RNN.

**S10. Different angular ranges and sampling geometries**


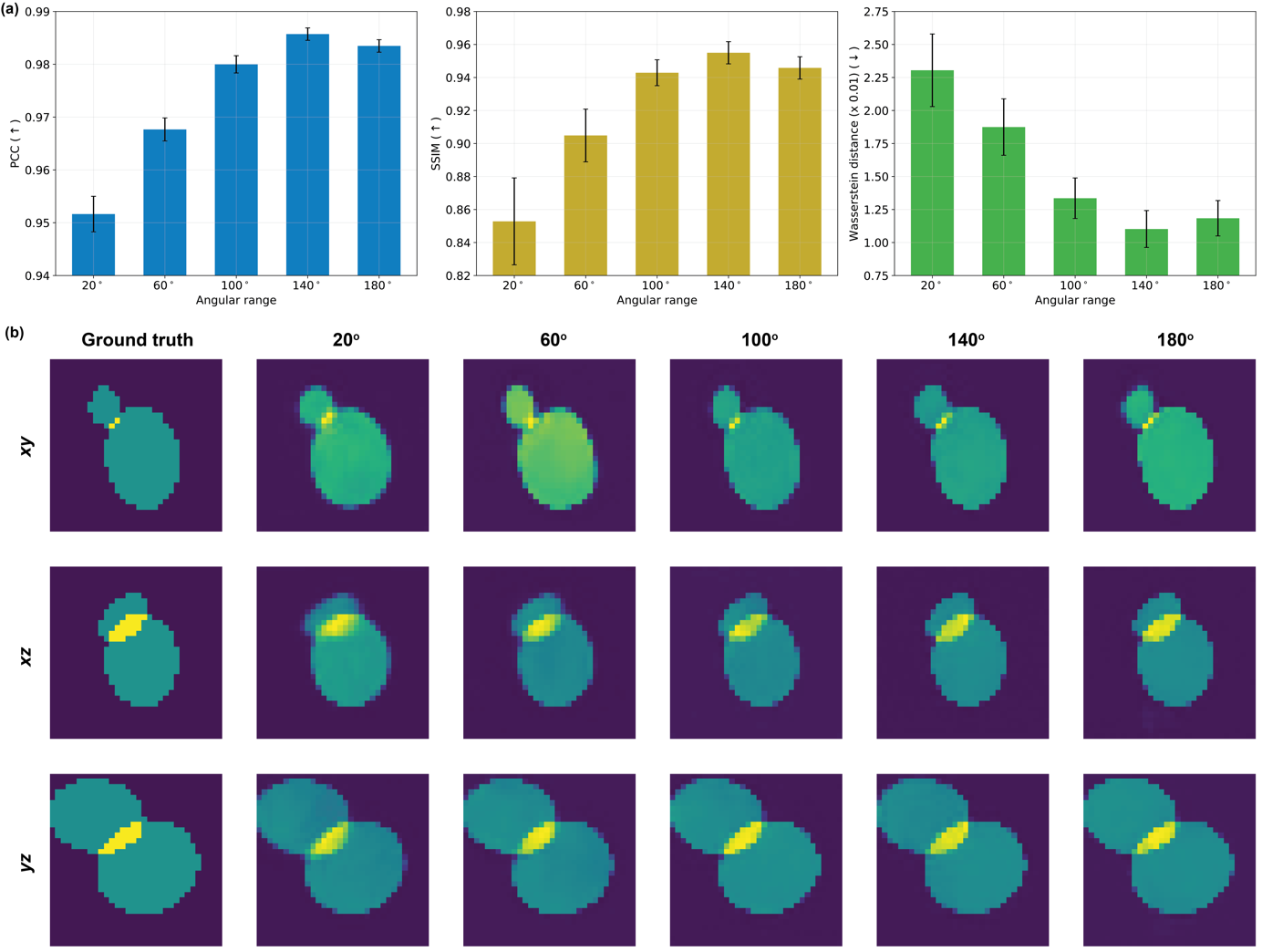


**Figure S11.** (a) Quantitative comparison of reconstructions based on different angular ranges using three different metrics (PCC, SSIM, Wasserstein distance), and (b) qualitative comparison of 2D cross-sections of reconstructions. The graphs show means and 95% confidence intervals.

In Figure S11, we fix the number of projections and photon budget for each projection to be 21 and 100 photons per pixel (purely Poissionian) and vary the angular ranges, *i.e.* 20, 60, 100, 140, and 180 degrees. Here, the projections are uniformly sampled within each angular range, and thus the first case represents a dense angular sampling within a limited angular range, and the last case a sparse angular sampling within a full angular range. As expected, a larger angular range with sparser angular sampling performs better than a smaller angular range with denser angular sampling.

**S11. Performance of RNN for objects with continuous profile**


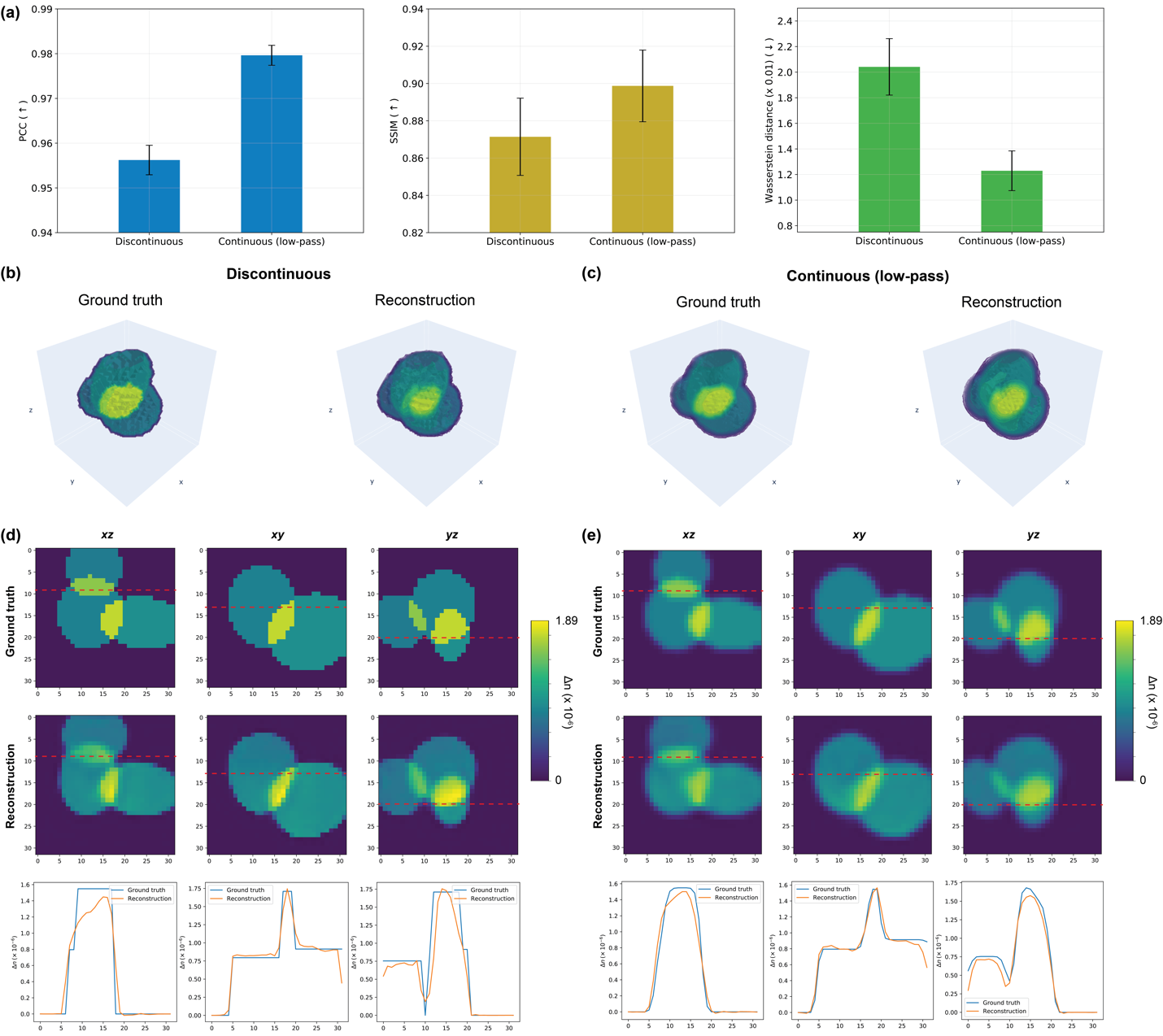


**Figure S12.** Comparison on performance of the proposed RNN under the weak scattering assumption on objects with binary and continuous Refractive Index (RI) profiles (a) quantitatively using three metrics (PCC, SSIM, and Wasserstein distance) and (b-e) qualitatively. In (e), red dotted lines show where the 1D cross-section profiles are referring to.

We demonstrate performance of the RNN architecture with objects whose Refractive Index (RI) is continuously distributed. Here, we assume ray optics approximation for simulation and negligible deflection angle of rays. To satisfy the assumption, the gradient of RI should be much less than 1. In the simulation, objects with discontinuous profiles are smoothened with a Gaussian kernel in order to reach $\left| \nabla n \right| \sim{10}^{-2} .$ In Figure S12, the performance with discontinuous and continuous objects is shown, and both quantitatively and visually, the continuous objects lead to better fidelity reconstructions.

**S12. Supplementary movies**

Three supplementary movies provide additional visualization on Figures 4 and 10 in the main manuscript. Supplementary movies 1 and 2 show the progression of weak scattering or projection tomographic reconstructions using simulated data. Ground truth and Filtered Backprojection (FBP)^3^ reconstructions are also displayed for comparison. Supplementary movies 3 and 4 show the progression of reconstructions using experimental data under strong scattering assumptions as the number of diffraction patterns $n$ and DWMA Approximants *m* entering the network increases, respectively. Ground truth, measurements, and Approximants are also provided for reference.

**References**

1. Bahdanau, D., Cho, K. & Bengio, Y., “Neural machine translation by jointly learning to align and translate,” *arXiv preprint* arXiv:1409.0473 (2014).

2. Vaswani, A. et al, “Attention is all you need,” in *Adv. Neural Inf. Process. Syst. (NIPS),* (2017), pp. 5998–6008.

3. Bracewell, R. N. & Riddle, A., “Inversion of fan-beam scans in radio astronomy,” *Astrophys. J.* 150, 427 (1967).

4. Goy, A. et al, “High-resolution limited-angle phase tomography of dense layered objects using deep neural networks,” *Proc. Natl. Acad. Sci*. 116, 19848–19856 (2019).
